# Vincristine-induced brain toxicity is reduced with prevention of peripheral axon degeneration in Sarm1 knockout mice

**DOI:** 10.1186/s40478-025-02171-0

**Published:** 2025-12-19

**Authors:** Jonas Yeung, Prisca Hsu, Jordan Mak, Ali Darbandi, Anne L. Wheeler, Rosanna Weksberg, Sharon L. Guger, Russell J. Schachar, Shinya Ito, Johann Hitzler, Brian J. Nieman

**Affiliations:** 1 Mouse Imaging Centre, Hospital for Sick Children, 25 Orde Street, Toronto, ON M5T 3H7 Canada; 2 Translational Medicine, Hospital for Sick Children Research Institute, Toronto, ON Canada; 3 Department of Medical Biophysics, University of Toronto, Toronto, ON Canada; 4 Neuroscience and Mental Health, Hospital for Sick Children Research Institute, Toronto, ON Canada; 5 Department of Physiology, University of Toronto, Toronto, ON Canada; 6 Nanoscale Biomedical Imaging Facility, Hospital for Sick Children, Toronto, ON Canada; 7 Department of Pediatrics, University of Toronto, Toronto, ON Canada; 8 Institutes of Medical Science, University of Toronto, Toronto, ON Canada; 9 Department of Psychology, Hospital for Sick Children, Toronto, ON Canada; 10 Department of Psychiatry (Emeritus), University of Toronto, Toronto, ON Canada; 11 Clinical Pharmacology and Toxicology, Hospital for Sick Children, Toronto, ON Canada; 12 Department of Pharmacology and Toxicology, University of Toronto, Toronto, ON Canada; 13 Division of Haematology/Oncology, Hospital for Sick Children, Toronto, ON Canada

**Keywords:** SARM1, Axon degeneration, Vincristine, Peripheral neuropathy, Chemotherapy, MRI

## Abstract

Vincristine is an essential chemotherapy agent administered for various pediatric cancers including acute lymphoblastic leukemia (ALL). While multi-agent chemotherapy for pediatric ALL is highly curative, with survival approaching 95%, it carries the risk of irreversible neurocognitive late effects. Vincristine is known to cause peripheral neuropathy, but its relationship to brain toxicity remains understudied. We investigated vincristine-mediated brain toxicity in young mice lacking *Sarm1*, a gene whose deletion protects against vincristine-induced peripheral neuropathy. Littermate wildtype and knockout mice were randomly assigned to saline or vincristine groups. In vivo MRI was performed from childhood to early adulthood to measure brain structure volumes, followed by ex vivo diffusion tensor imaging (DTI) to assess microstructural changes. In a separate cohort, electron microscopy (EM) quantified axon morphology in the sciatic nerve and corpus callosum. Vincristine induced significant volume reduction across the brain, while *Sarm1* knockout reduced loss in both grey and white matter. Several regions, including the amygdala and dentate gyrus, showed near-complete recovery in knockouts. DTI revealed limited changes with no genotype differences. EM demonstrated vincristine-induced axon morphology alterations in wildtype mice in both the sciatic nerve and corpus callosum. *Sarm1* knockout rescued sciatic nerve morphology but not corpus callosum axons. These findings suggest that SARM1-mediated peripheral axon damage may contribute to vincristine-induced brain volume deficits, whereas brain axons may be affected through distinct, SARM1-independent mechanisms. These results suggest a link between vincristine-induced peripheral axon damage and alterations in brain development, with implications for neurocognitive deficits experienced by ALL survivors. Our results suggest that mitigating vincristine-induced peripheral neuropathy may also help reduce neurocognitive deficits in pediatric patients undergoing vincristine treatment.

**Supplementary Information:**

The online version contains supplementary material available at 10.1186/s40478-025-02171-0.

## Introduction

Vincristine (VCR) is a chemotherapy agent used to treat many pediatric cancers including leukemias, lymphomas, sarcomas, and brain tumors [1]. For example, VCR is administered at every stage of treatment for pediatric acute lymphoblastic leukemia (ALL), the most prevalent childhood cancer with peak incidence between 3 and 5 years of age [2, 3]. While ALL treatment is highly curative, resulting in almost 95% five-year survival rate [3, 4], it is associated with the risk of persistent neurocognitive deficits among survivors [5–8]. Approximately 40–60% of survivors experience cognitive issues [9, 10] affecting their working memory, processing speed, and attention [9, 11–14]. With treatment commencing at such a young age, these side effects have lifelong ramifications as many survivors show decreased academic and employment success, and reduced quality of life [15–18]. Due to the complexity of ALL treatment and the potential for effects from cancer itself, it is advantageous to use preclinical models to isolate components of treatment for individual study of their contributions to brain toxicities. Notably, a mouse model of childhood leukemia treatment, in which a number of chemotherapy agents were tested, determined that VCR treatment resulted in widespread brain volume deficits, mirroring the phenotype observed in childhood ALL survivors [19, 20]. This makes VCR a candidate for further investigation into its potential role in the development of neurocognitive side effects among ALL survivors.

Side effects of VCR treatment are clinically well recognized, and primarily associated with symptoms in the peripheral nervous system [21]. As a vinca alkaloid, the anti-cancer mechanism of action for VCR is to irreversibly bind to microtubules and spindle proteins to block mitosis during metaphase [22]. However, VCR can exert off-target effects by binding to microtubules within neuronal axons and initiate axon degeneration [23]. In the peripheral nervous system, this manifests as vincristine-induced peripheral neuropathy (VIPN) affecting sensory, motor, and autonomic nerves. Nearly all children treated with VCR experience VIPN, which is most often observed clinically as numbness, tingling, and painful sensation in the hands and feet [24, 25]. Peripheral neuropathy is one of the most acute dose-limiting side effect of VCR. However, VCR has not traditionally been associated with toxicity in the brain, perhaps because VCR itself does not efficiently penetrate the blood-brain barrier [26–28]. Given the observation that VCR treatment induced significant brain volume loss in a murine model [19], we sought to investigate whether VCR-induced axon degeneration is linked to brain toxicity in a mouse model.

Recent studies have demonstrated that SARM1 (sterile alpha and TIR motif containing protein 1) is an important mediator for axon degeneration in the central and peripheral nervous system [29–31]. SARM1 is primarily expressed in neurons and remains in an inactive state under typical healthy conditions. Upon axon injury, SARM1 can be activated and result in the rapid breakdown of NAD^+^, leading to axon degeneration [32, 33]. Geisler et al. [34] observed that VIPN is prevented by deletion of the gene *Sarm1* in 20-week-old mice. The ability to alter axon degeneration by genetic manipulation of *Sarm1*—particularly in the peripheral nervous system where VCR has a significant effect—provides the opportunity to investigate the role of peripheral axon degeneration in VCR-induced brain toxicity.

Here, we use genetically modified *Sarm1* knockout mice and MRI to evaluate whether limiting axon degeneration protects the brain from VCR-induced toxicities. In vivo three-dimensional MRI provides a translatable and whole-brain approach to study the effects of VCR-induced toxicity on the brain throughout development [19, 35]. We show that *Sarm1* knockout partially mitigates VCR-induced neuroanatomical deficits. Diffusion tensor imaging (DTI) analysis revealed limited effects of VCR on water diffusivity in brain tissue, with no difference between wildtype and *Sarm1* knockout mice. Electron microscopy image analysis revealed that *Sarm1* knockout mitigates the effects of VCR on peripheral axons, but that axons in the brain (as evidenced in the corpus callosum) are affected regardless of *Sarm1* status. These findings suggest that SARM1-mediated axon degeneration processes may not be directly involved in the brain, while VIPN could be the major contributor of neuroanatomical deficits as detected by MRI. The experimental demonstration of a link between peripheral axon degeneration and VCR-induced brain toxicity supports the hypothesis that symptoms of VIPN raise risk for neurocognitive deficits in ALL patients [36] and suggest that interventions that address VIPN may also have a positive impact on long-term brain outcomes in ALL survivors.

## Methods

### Mice and experimental timeline

**Fig. 1:**
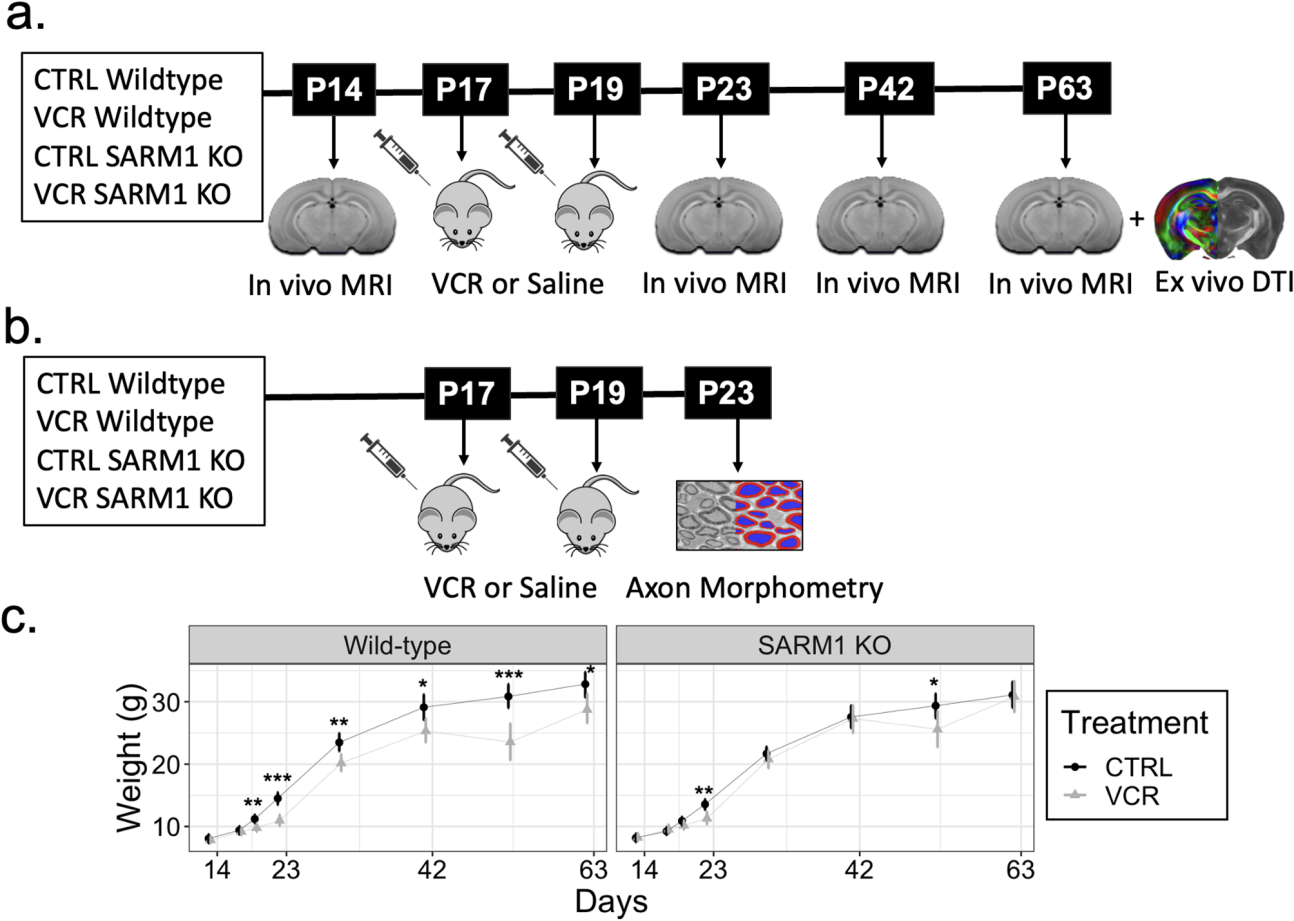
**A** Experimental timeline for MR imaging. Four groups of mice were employed, with all combinations of treatment (VCR or CTRL) and genotype (wildtype or *Sarm1* KO). Mice were imaged in vivo at P14, P23, P42 and P63. At endpoint, brains were also scanned ex vivo with DTI. VCR or saline injections occurred at P17 and P19. **B** Experimental timeline for transmission electron microscopy to measure axon morphometrics. After treatment, the sciatic and corpus callosum were harvested at P23. **C** Mouse weights for each group. VCR treatment induced significant weight loss in wildtype mice. Weight loss was significantly reduced in *Sarm1* KO mice. * *p* < 0.05, ** *p* < 0.01; *** *p* < 0.001

All procedures were approved by the Centre for Phenogenomics Animal Care (TCP) Committee. N3 C57B6/J; CD1 hybrids of both sexes were used for breeding in all experiments. This hybrid background was preferred because it more nearly matched the original CD-1 strain as characterized by Spencer Noakes et al. [19] and because the C57BL/6J mice showed higher and variable sensitivity to VCR compared to CD1 and N3 hybrids. These hybrids were produced by breeding male C57BL/6J *Sarm1* homozygous constitutive KO (*Sarm1* KO) mice obtained from Jackson Laboratory (B6.129 × 1-Sarm1^tm1Aidi^/J, #018069) with female wildtype CD-1 mice (TCP, in-house). The *Sarm1* gene is disrupted in exons 3–6, resulting in a germline constitutive loss of SARM1 function [37]. Heterozygous *Sarm1* F1 hybrid male mice were twice consecutively bred with CD-1 mice to produce N3 offspring heterozygous for *Sarm1* deletion. Mice heterozygous for *Sarm1* were bred with each other to generate *Sarm1* homozygous constitutive knockout (*Sarm1* KO) and wildtype (WT) mice in the same litter. Mouse genotypes were obtained by tail biopsy samples using real-time PCR with specific probes for each allele (Transnetyx, Cordova, TN). Littermates of both genotypes were randomized into saline- (CTRL) and VCR-treated groups. Mice were treated at postnatal day (P)17 and P19 in accordance with a previous mouse model of childhood leukemia [19]. Mice were scanned with MRI at P14 (pre-treatment), P23, P42, and P63 to observe brain development from childhood to early adulthood. Ex vivo DTI was also performed after the last scan timepoint at P63. The experimental timeline for scanning is summarized in Fig. 1A. The study consists of four groups: CTRL WT, VCR WT, CTRL *Sarm1* KO, and VCR *Sarm1* KO. Each grouped consisted of approximately 15 mice split equally between sexes. The specific number of mice per imaging timepoint is shown in Supplementary Table 1.

### Vincristine injections

VCR (vincristine sulphate, Hospira healthcare) or saline was administered intravenously via retro-orbital injection at P17 and P19 with half of the total dose given each day. Delivery route and total VCR dose of 1.05 mg/kg were selected based on the mouse model of childhood ALL treatment described in Spencer Noakes et al. [19]. For treatment, mice were anesthetized with 2% isoflurane and then transferred to a nose cone on top of a heated pad to be injected. All injections were delivered to the right orbital sinus. After the procedure, mice were moved to a heated cage to recover. Following treatment, weights were measured 24-hours before each MR imaging timepoints and as well as on P30 and P52.

### In vivo magnetic resonance imaging and data processing

In vivo MR images were acquired with a 7-T MRI scanner (Bruker BioSpin, Ettlingen, Germany) equipped with four cryocoils to image four mice simultaneously [38]. Scans were performed with the following settings: T1-weighted, 3D-gradient echo sequence, 75 μm isotropic resolution, TR = 26 ms, TE = 8.25 ms, flip angle = 26°, field of view = 25 × 22 × 22 mm, and matrix size = 334 × 294 × 294. Mice received an intraperitoneal injection of manganese chloride contrast at a dose of 0.4mmol/kg 24-hours prior to imaging [39]. During imaging, mice were anesthetized with 1% isoflurane and monitored in real-time for respiration based on data collected from the scanner [40].

Anatomical differences between images were quantified by employing an automatic registration pipeline within the Pydpiper toolkit to align images into a consensus average space [41]. A pre-segmented atlas with 183 brain regions was registered onto each image to obtain volumetric measurements using the MAGet brain algorithm [42–46].

### Ex vivo diffusion tensor imaging and data processing

Following the final in vivo MRI scan at P63, the mice were sacrificed, and their brains were fixed using established protocols [47, 48]. Ex vivo MR images were acquired in the same 7-T MRI scanner scanner (Bruker BioSpin, Ettlingen, Germany). Eight samples were imaged in parallel using a custom made 8-coil solenoid array. The diffusion tensor data were acquired with the following settings: 3D-segmented EPI, 80 μm isotropic resolution, TR = 300 ms, TE = 44 ms, field of view = 25.6 × 19.2 × 19.2 mm, 20 segments, and matrix size = 320 × 240 × 240, total acquisition time = 14.4 h. Six non-diffusion weighted (b = 0 s/mm^2^) and 30 diffusion weighted (b = 2000 s/mm^2^) images were acquired for each sample.

### Ex vivo DTI registration and data processing

A rigid registration was performed to align the 6 non-diffusion and 30 diffusion images in the same space for each brain sample. A convex optimization algorithm [49] was employed to solve for the eigenvectors and eigenvalues to obtain the DTI metric maps for fractional anisotropy (FA), mean diffusivity (MD), axial diffusivity (AD), and radial diffusivity (RD). Samples were excluded based on the criteria that the direction images were not within 1.5 standard deviation of the mean directional images across all samples. The average non-diffusion weighted images for all samples were aligned into a consensus average space using the Pydpiper toolkit [41]. Each DTI metric map was transformed into the consensus average space. A pre-segmented atlas with 185 labels was registered onto the consensus average using the MAGeT algorithm [46] to calculate the average DTI metric for each brain region. The direction encode color (DEC) map was displayed on FSLeyes (FSL version 6.0.7) by displaying the average principal eigenvector in a 3-directional vector RGB image with the intensity modulated by the average FA.

### Transmission electron microscopy

To examine axon morphology, a separate cohort of mice were prepared using the same treatment paradigm and were sacrificed at P23 for tissue harvesting (*n* = 5 per group, approximately 2–3 of each sex). The experimental timeline is summarized in Fig. 1B. Mice were perfused and fixed using established protocols [47]. The right sciatic nerve was identified, and the distal sciatic nerve was dissected out using the trifurcation into the tibial, sural, and peroneal nerves as a landmark. The distal sciatic nerve was stored in electron microscopy (EM) specific fixative (2.5% glutaraldehyde, 0.02% calcium chloride, 4% PFA in 0.1 M cacodylate buffer) for approximately 24-hours. Brains were collected and stored in EM fixative using established protocols for one day minimum. The anterior corpus callosum was dissected from each brain and stored in EM fixative for approximately 24-hours [50]. All samples were then stained with 1% Osmium tetraoxide for 90 min, then dehydrated, embedded, sectioned into 50 nm cross-sections (ultramicrotome EM UC7, Leica Microsystems). TEM grids were post stained with 2% Uranyl acetate and 1% lead citrate. Grids were imaged using a transmission electron microscope (HT7800 TEM, Hitachi) at the Nanoscale Biomedical Imaging Facility (SickKids, Toronto, Ontario, Canada). Images for segmentation were acquired at 700x and 3000x magnification for the sciatic nerve and corpus callosum, respectively.

### Electron microscopy image analysis

Axons and myelin were segmented using the AxonDeepSeg (version 4.1.0) semi-automatic pipeline from the Napari software (version 0.4.19) [51]. Two images per sample with different fields of view were collected for morphological analysis with the exception of 1 image where two field of views were not possible. Morphometrics were computed for each image, generating measurements including axonal area and myelin area. Morphometrics from images of the same mouse were pooled and all measurements were binned to generate histogram distributions of axon and myelin areas. Statistical significance between treatment and genotype groups was assessed within each bin using a two-tailed t-test.

### Statistical analysis

Group comparisons were made with a linear model for electron micrographs and DTI metric analysis. The model consisted of terms for genotype, treatment, and its interactions. For longitudinal in vivo MRI, brain structure volumes were fit using a linear-mixed effects model. The full statistical model is shown explicitly in the Supplementary Methods. The model includes fixed effects coefficients for each scan age, VCR treatment, *Sarm1* KO, VCR treatment and *Sarm1* KO interaction, and their respective linear slopes. A random effect was included to account for individual mouse variability. The same statistical model was used to evaluate relative structural volumes (obtained by normalizing each brain structure volume by total brain volume in each mouse). *p*-values were obtained by using the Satterthwaite approximation [52]. Uncorrected *p*-values are simply referred to as “*p*-values”. The false discovery rate (FDR) was used to correct for multiple comparisons, which are reported as “q-values” [53]. Sex and treatment interactions were excluded as a term in the main model after determining that there were no statistically significant sex differences. All statistics are reported as mean ± 95% confidence intervals unless otherwise stated. All statistical analyses were performed using R (version 3.6.3).

## Results

### Vincristine treatment causes weight loss that is mitigated by ***Sarm1*** knockout

Mice weights were measured 24-hours before each imaging timepoint, during treatment, and at P30 and P52 (Fig. 1C). VCR induced observable weight loss by P19 (childhood equivalent), two days after the first VCR treatment, in wildtype mice (− 12% ± 8%, *p* < 0.01). Weight deficiency persisted for VCR wildtype mice until endpoint at P63. *Sarm1* KO mice were protected from VCR-induced weight loss at most timepoints, exhibiting small weights at P22 (− 16% ± 11%, *p* < 0.01) and transiently at P52 (− 13% ± 13%, *p* = 0.05). For both wildtype and *Sarm1* KO mice treated with VCR, we observed a late-appearing and transient weight reduction in VCR-treated mice at P52, recapitulating an observation by Spencer-Noakes, et al. [19] Overall, however, *Sarm1* KO largely mitigated VCR-induced weight loss, suggesting its ability to reduce the systemic side effects of VCR. While weight differences were observed, no evident signs of distress were observed in VCR-treated mice. Although, qualitatively, slight trembling of the hind limbs was observed when the VCR-treated mice were handled.

### Vincristine induces widespread volume loss in the brain

**Fig. 2:**
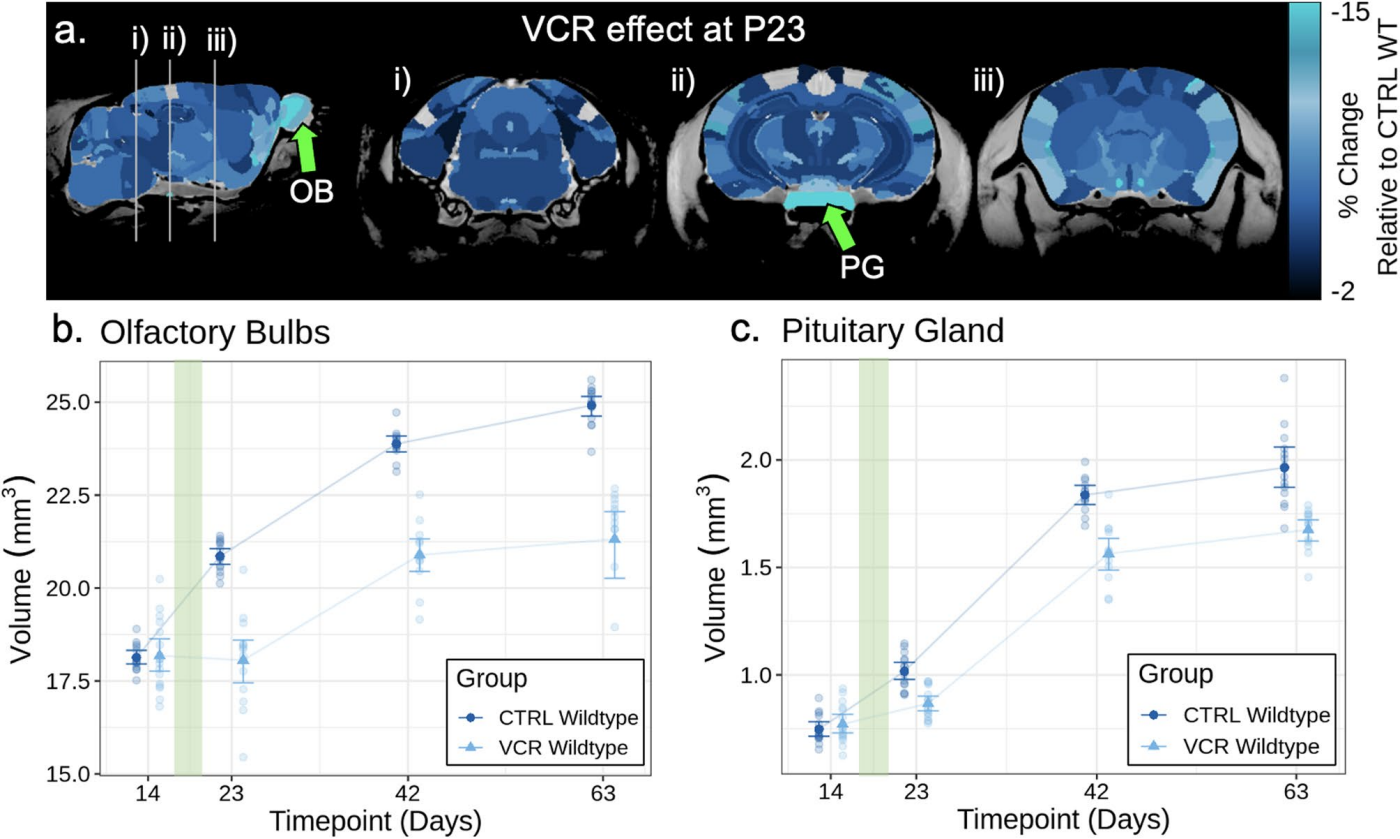
**A** Neuroanatomical maps of the mouse brain highlighting structures that were significantly affected by VCR treatment at P23 (q < 0.1). Significant volume loss was observed in most brain structures, with the most noticeable changes in the olfactory bulbs and pituitary gland. All colored structures exhibit statistically significant volume loss (q < 0.1). **B** and **C** Longitudinal volumes are plotted for olfactory bulbs and the pituitary gland in CTRL and VCR wildtype mice from P14 to P63. Following VCR treatment, at P17 and P19 visualized vertically in green, there is a significant volume loss observed at P23 relative to CTRL in both the olfactory bulbs (*p* < 0.001) and the pituitary gland (*p* < 0.001). Solid points and whiskers represent mean structure volume and bootstrapped 95% confidence intervals, respectively, after removal of fitted random effects. Groups and data points were jittered horizontally for visualization. OB, olfactory bulbs. PG, pituitary gland

We observed widespread volume loss in the brains of wildtype mice after VCR treatment. VCR-induced volume loss at P23 was quantified by normalizing to CTRL WT volume for each structure to compute a percentage change (Fig. 2A). At P23, VCR induced volume loss in 165 out of 183 brain structures. Out of the 165 evaluated structures, 122 of 134 (91%) grey matter structures and 33 of 39 (85%) white matter showed volume loss. The olfactory bulbs (OB) and pituitary gland (PG) were the two structures exhibiting the greatest VCR-induced change at P23, with 13% ± 4% (*p* < 0.001) and 16% ± 8% (*p* < 0.001) smaller volumes than CTRL, respectively. The longitudinal volumes of both structures are provided in Fig. 2B–C. The volume deficits of the OB and PG worsen over time, similar to what is observed after cranial radiation [54]. A comprehensive description of the statistical results by structure is provided in Supplementary Table 2. Although statistically insignificant, the effect of VCR for each sex, as assessed by an exploratory linear mixed-effects model, is presented in Supplementary Fig. 1. Due to significant widespread changes in brain volumes, we also evaluated relative structural volumes (by normalizing each structure to total brain volume for each mouse) to determine whether specific regions were disproportionately affected beyond global brain size reductions (Supplementary Fig. 2A). At P23, VCR induced relative structure volume change in 22 out of 183 brain structures. Consistent with absolute volumes, significant relative volume reductions were also observed in the olfactory bulbs (− 13% ± 4%, *p* < 0.001) and pituitary gland (− 10% ± 6, *p* < 0.01).

### ***Sarm1*** knockout reduces early vincristine-induced total brain volume loss

**Fig. 3:**
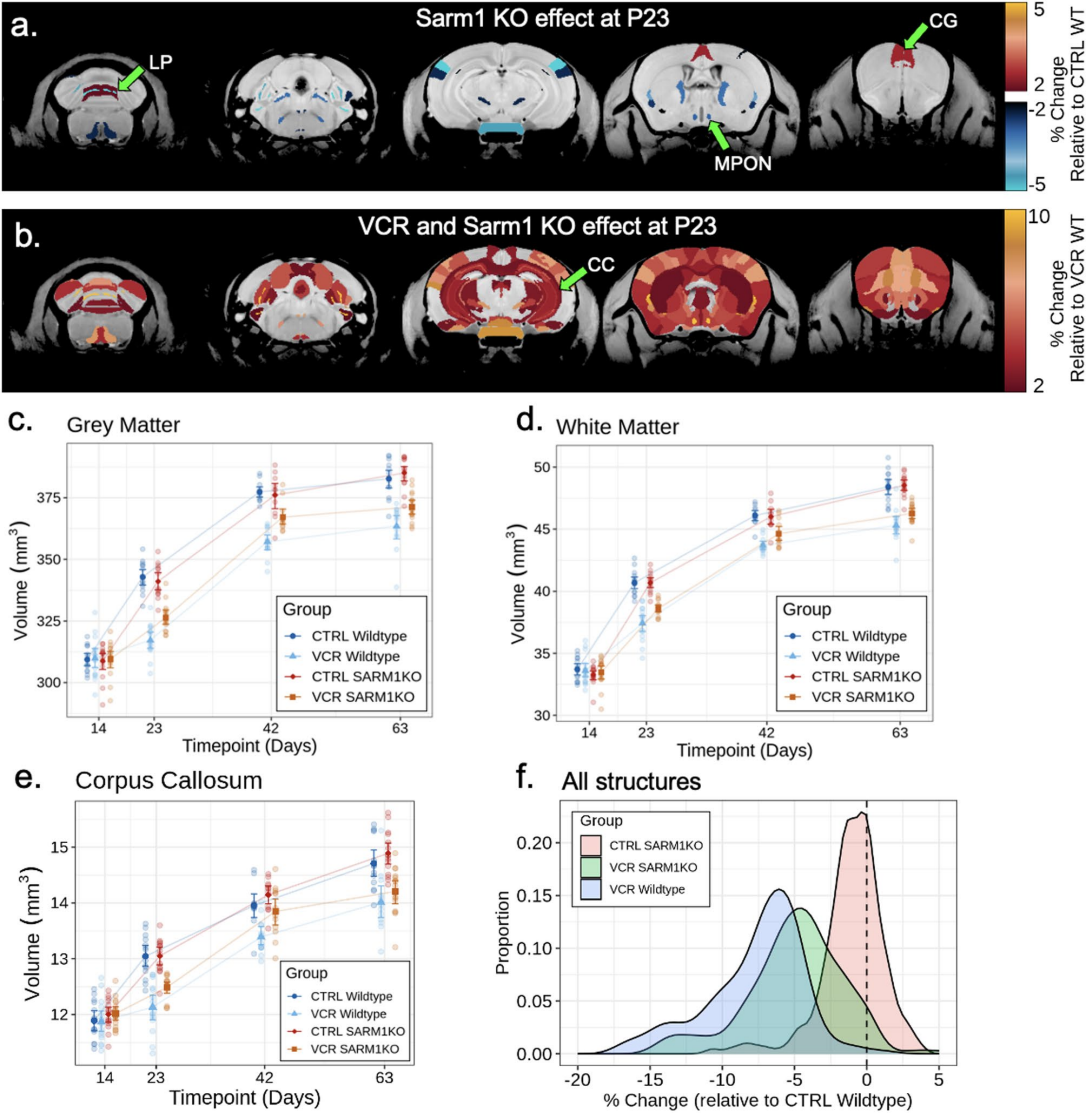
The effects of *Sarm1* KO on the brain and its interaction with VCR treatment. **A** Coronal neuroanatomical maps at P23 show minimal effects of *Sarm1* KO in saline-treated mice, with few structures affected. **B** In VCR-treated mice, *Sarm1* KO reduced structural loss compared to VCR-treated wildtypes, indicating neuroprotection. **C**, **D** Grey matter (GM) and white matter (WM) volumes decreases in VCR-treated wildtypes (GM: − 7.1% ± 1.8%, *p* < 0.001; WM: −7.6% ± 2.2%, *p* < 0.001) but were partially rescued in *Sarm1* KO mice (GM: +3.0% ± 2.0%, *p* < 0.01; WM: +3.2% ± 2.6%, *p* < 0.05). No GM or WM differences occurred between saline-treated wildtype and *Sarm1* KO mice. **E** The corpus callosum (CC), the largest white matter structure in the brain, also showed a modest improvement to VCR treatment when knocking out *Sarm1* (+ 2.3% ± 3.0%, *p* = 0.142). Points and whiskers represent mean structure volume and bootstrapped 95% confidence intervals, respectively, after removal of fitted random effects. Groups and data points were jittered for visualization. **F** The percent volume change for all 183 brain structures in the atlas were binned into a density histogram. Histograms were generated for the three other group using the saline-treated (CTRL) wildtype group as the baseline. The CTRL *Sarm1* KO distribution exhibited slight left-shift (red curve, mean= − 0.98% ± 0.32%, *p* < 0.001) as some structures were smaller compared to wildtype mice. Structures in the VCR-treated wildtype mice exhibited a significant widespread loss (blue, mean = − 8.2% ± 0.6%, *p* < 0.001) but is partially improved when Sarm1 was knockout (orange, mean = − 5.0% ± 0.5%, *p* < 0.001). LP, lobule VIII: pyramis; MPON, medial preoptic nucleus; CG, cingulate cortex 32; CC, corpus callosum

Next, we assessed the effect of knocking out *Sarm1* in saline-treated mice to observe the effects of *Sarm1* KO relative to wildtype at P23. There were no total grey matter nor white matter differences (*p* > 0.05) between *Sarm1* KO and wildtype saline-treated mice. We found only a few brain structures that trended to be different due to *Sarm1* KO (Fig. 3A): the medial preoptic nucleus (− 2.9% ± 8.1%, *p* = 0.48), lobule 8: Pyramis (2.0% ± 4.4%, *p* = 0.37), and cingulate cortex area 32 (2.5% ± 4.1%, *p* = 0.22). Nonetheless, a density histogram of all structures shows an overall trend to a small volume decrease in *Sarm1* KO mice (Fig. 3F, red curve, all structures mean= − 0.98% ± 0.32%, *p* < 0.001). *Sarm1* KO had minimal effect on relative structural volumes (Supplementary Fig. 2B).

To assess whether *Sarm1* KO improved brain volume outcomes after VCR treatment, we calculated the degree of rescue for each structure in the brain atlas (determining the percent change by normalizing the VCR-*Sarm1* KO interaction term by the absolute volume in VCR-treated wildtype mice). Widespread rescue in response to VCR treatment was observed in *Sarm1* KO mice as early as P23 (early adolescent equivalent), with mitigation of volume loss compared to VCR treatment in wildtype mice. Out of 165 structures affected by VCR in wildtype mice, 27 of them were found to be improved in *Sarm1* KO mice at P23 (q < 0.1). In Fig. 3B, we show all structures at P23 with at least 2% change from the VCR and *Sarm1* KO interaction term normalized by the VCR WT structure volumes, which represents 128 structures. Reduced VCR-induced volume loss in *Sarm1* KO mice was observed in total grey matter (Fig. 3C, + 3.0% ± 2.1% compared to VCR WT, *p* < 0.01) and total white matter (Fig. 3D, + 3.2% ± 2.6% compared to VCR WT, *p* < 0.05). The corpus callosum is also shown in Fig. 3E, representing the largest white matter structure in the brain (+ 2.3% ± 3.0% compared to VCR WT, *p* = 0.142). To compare the distribution of volume changes in VCR-treated *Sarm1* KO mice compared to wildtype mice at P23, density histograms representing all structures in the brain are plotted in Fig. 3F. The curve for VCR-treated *Sarm1* KO mice (orange) exhibits a shift to larger volumes (mean = -5.0% ± 0.5% from vehicle WT, *p* < 0.001) compared to VCR-treated wildtype (blue, mean = − 8.2% ± 0.6% from vehicle WT, *p* < 0.001). This highlights an overall pattern of improvement early after treatment due to *Sarm1* KO compared to wildtype. Qualitatively, *Sarm1* KO mitigated VCR-induced relative volume loss (Supplementary Fig. 2C). Although statistically insignificant, the effect of VCR and *Sarm1* KO with sex, as assessed by an exploratory linear mixed-effects model, is presented in Supplementary Fig. 1.

### ***Sarm1*** knockout rescues long-term VCR-induced brain volume loss

**Fig. 4:**
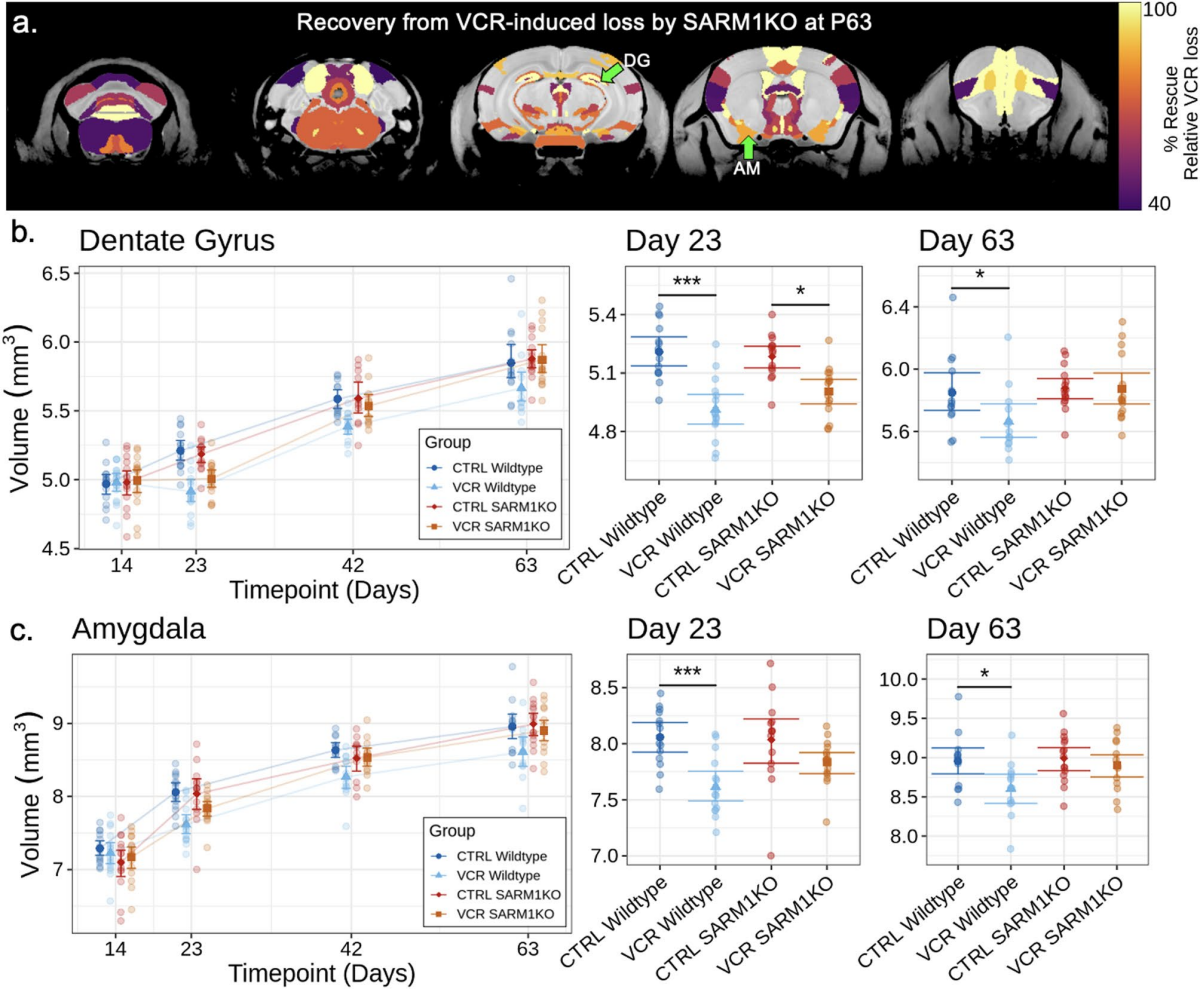
Structures in the brain that exhibited significant rescue by P63 from VCR treatment after knockout of *Sarm1*. **A** Neuroanatomical maps showing the percentage rescue, calculated by normalizing the volume improved to VCR-induced loss. Several structures achieved nearly full rescue from volume loss due to VCR treatment, such as the dentate gyrus (DG) and amygdala (AM). **B** and **C** The dentate gyrus and amygdala volumes over time with individual timepoint panels at right for P23 and P63. Both structures achieved significant rescue by P63. Points and whiskers represent mean structure volume and bootstrapped 95% confidence intervals, respectively, after removal of fitted random effects. Groups and data points were jittered in the time course curves for the purpose of visualization. * *p* < 0.05, *** *p* < 0.001. DG, dentate gyrus; AM, amygdala

To observe persistent long-term effects, we evaluated the degree of rescue after VCR treatment due to *Sarm1* KO at early adulthood (P63). Percent rescue is shown in Fig. 4A, computed by normalizing the improvement observed in *Sarm1* KO mice to the VCR-induced volume loss in wildtype mice (e.g., a rescue of 100% means a return to vehicle treated volume with no residual VCR-induced volume loss). All structures displayed with a color overlay in Fig. 4A exhibited at least a 40% rescue (no threshold) and were significantly decreased in volume after VCR treatment (q < 0.1). Out of 165 structures significantly affected by VCR treatment, 84 were highlighted in the brain to exhibit a recovery of at least 40%. A few structures exhibited a complete or near complete rescue. For example, the dentate gyrus (Fig. 4B) exhibited initial VCR-induced volume loss slightly less than wildtypes (rescue of 33% at P23) but by early adulthood (P63) experienced a complete volume rescue. The rescue in the dentate gyrus is particularly interesting given that chemotherapy has been reported to impair neurogenesis in the dentate gyrus with implications for memory [55]. Similarly, the amygdala (Fig. 4C) showed an initial P23 rescue of 70% followed by an improved P63 rescue of 84%. The percent rescue for all highlighted structures in Fig. 4A is shown in Supplementary Table 2.

### Vincristine slightly altered water diffusion in wildtype and ***Sarm1*** KO mice

**Fig. 5:**
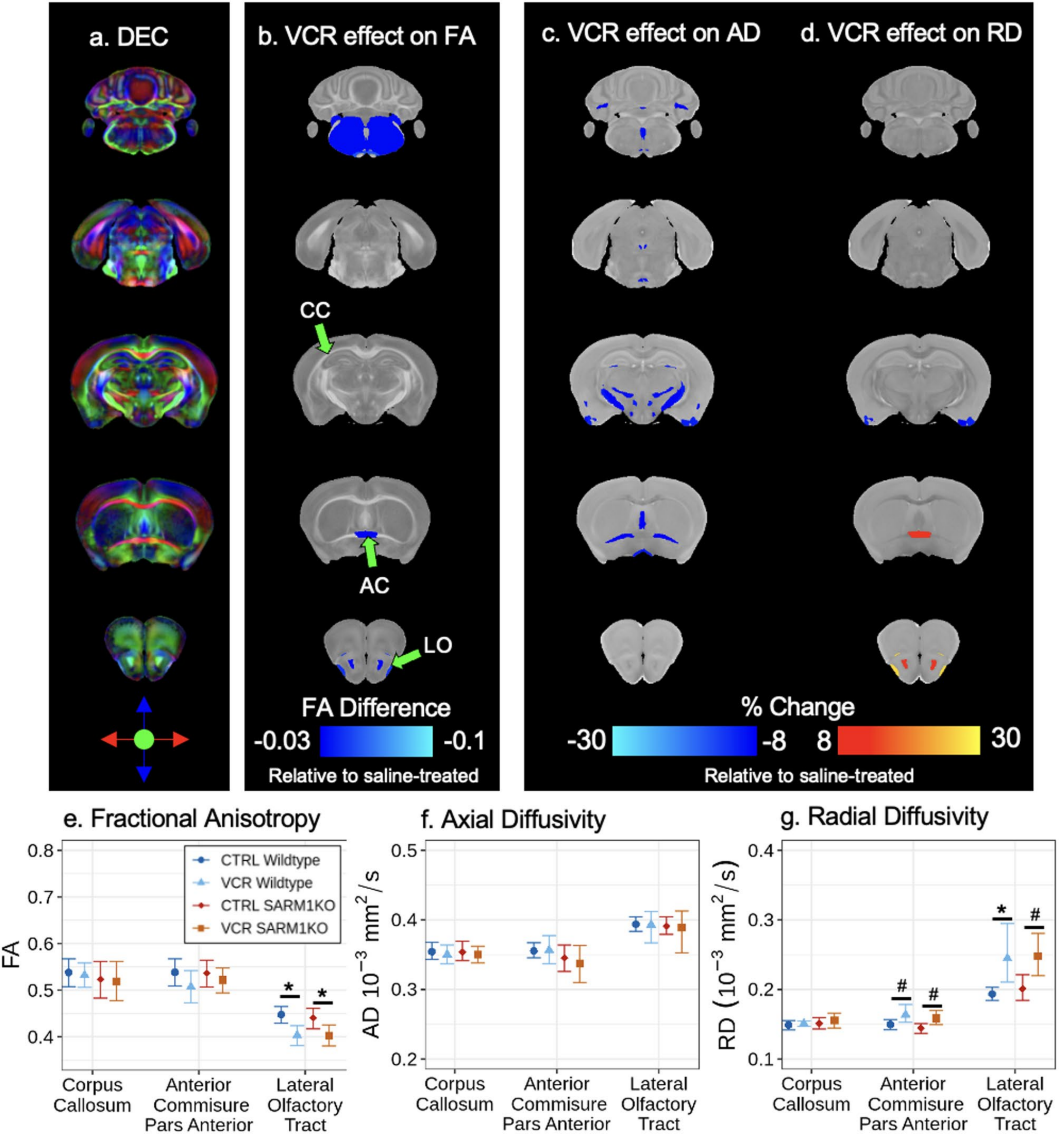
VCR effect on FA, AD, and RD in wildtype and *Sarm1* KO mice at P63. **A** The average diffusion encoded colour (DEC) map of the mouse brain. Directionality is encoded by color based on the principal eigenvector with intensity modulated by FA. Rostral and caudal directions are shown in green. Dorsal and ventral directions are shown in blue. Lateral directions are shown in red. **B** Neuroanatomical maps highlighting regions that exhibited a minimum change of -0.03 in FA due to VCR. No changes were considered statistically significant (q > 0.1). **C** and **D** Neuroanatomical maps highlighting regions that exhibited a minimum magnitude of 8% change in AD or RD. No changes were considered statistically significant (q > 0.1). **E–G** The FA, AD, and RD between treatment and genotype groups in several white matter structures. Points and whiskers represent mean structure volume and bootstrapped 95% confidence intervals, respectively. # *p* < 0.1, * *p* < 0.05. CC, corpus callosum; AC, anterior commissure pars anterior; LO, lateral olfactory tracts

To characterize microstructure changes in the brain, we next assessed the effect of VCR on water diffusion by comparing DTI metrics at P63. Colour-coded maps representing the principal eigenvector (i.e., direction of greatest diffusion) are shown for an average image in Fig. 5A. After FDR correction, VCR did not produce any statistically significant alteration in FA, AD, nor RD in any of the 185 structures for wildtype nor *Sarm1* KO mice (q > 0.1). To highlight the largest observed trends for completeness, we visualize maps (Fig. 5B–D) and plot derived diffusion metrics for several white matter structures (Fig. 5E–G). At a threshold uncorrected for multiple comparisons, VCR induced a decrease in FA for the lateral olfactory tracts in both wildtype (− 0.044 ± 0.034, *p* < 0.05) and *Sarm1* KO (-0.037 ± 0.032, *p* < 0.05). Though AD showed limited changes in white matter structures in VCR-treated mice, RD results tended to be increased in the anterior commissure pars anterior (WT: 9.3% ± 10.2%, *p* = 0.075; KO: 9.6% ± 10.3%, *p* = 0.066) and the lateral olfactory tracts (WT: 26.4% ± 25.2, *p* < 0.05; KO: 23.2 ± 23.4%, *p* = 0.052). Mean diffusivity (MD) was also shown to be statistically insignificant for all structures after multiple comparisons (Supplementary Fig. 3). Overall, we conclude that diffusion metrics were not significantly altered by VCR, and any sub-threshold trends showed that VCR induced similar alterations in both wildtype and *Sarm1* KO mice.

### ***Sarm1*** knockout protects peripheral axons from VCR-induced morphological alterations but not axons in the brain

Because *Sarm1* knockout is constitutive, it remains unclear whether the protection against VCR-induced brain volume deficits is due to the absence of SARM1-mediated processes in the brain itself or a downstream effect of preventing VIPN. To evaluate this, we assessed axon morphology to look for signs of vincristine-induced axon alterations in both the central and peripheral nervous system in a separate cohort of mice.

**Fig. 6:**
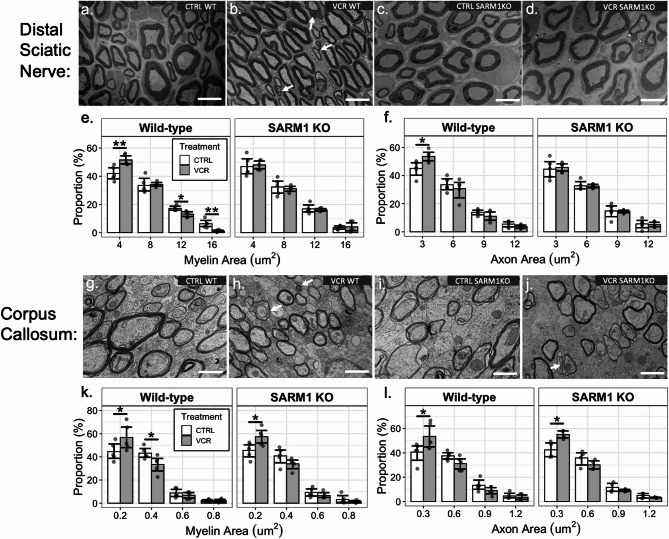
Axon morphology in the distal sciatic nerve and corpus callosum (*n* = 4–5 per group). Myelin and axonal areas were segmented **A–D** TEM photomicrographs of peripheral axons in the distal sciatic nerve. Alterations in axon morphology seemed more apparent in VCR WT mice (white arrows) compared to other groups. Scale bar = 5 μm. **E** and **F** Myelin and axon area distribution of the sciatic nerve for wildtype and *Sarm1* KO mice treated with saline or VCR. Axon and myelin distribution in wildtype VCR-treated mice exhibited a left shift with smaller areas, whereas *Sarm1* KO axon size distribution exhibited no detectable alterations in morphology from VCR treatment. Scale bar = 1 μm. **G**–**J** TEM photomicrographs of white matter tracts in the corpus callosum. White arrows point to axon morphology that were deemed irregular shaped. **K** and **L** Myelin and axon area distribution of the corpus callosum for wildtype and *Sarm1* KO mice treated with saline or VCR. Axon and myelin size distribution for both wildtype and *Sarm1* KO mice were altered from VCR treatment, exhibited smaller areas. Whiskers represent bootstrapped 95% confidence intervals. Data points were jittered for visualization. * *p* < 0.05, ** *p* < 0.01

Previous reports have identified an VCR-induced change in axon size distribution that is linked with a VIPN-associated phenotype [34]. We therefore examined axon size distribution in a representative peripheral nerve, the distal sciatic nerve. This nerve was selected for analysis as it is straightforward to locate and anticipated to be among the most susceptible to VCR damage due to its length [56]. TEM photomicrographs were obtained of the cross section of the distal sciatic nerve. The distal sciatic nerve from wildtype (Fig. 6A) and *Sarm1* KO (Fig. 6C) mice exhibited no appreciable difference in the distribution of myelin (*p* > 0.05) nor axonal area (*p* > 0.05) under normal conditions. In VCR-treated wildtype mice, axons exhibited alterations in morphology (Fig. 6B) with a higher proportion of smaller axons and myelin (Fig. 6E and F). In contrast, VCR-treated *Sarm1* KO mice are not different from vehicle-treated mice (Fig. 6D). We observe a left shift in axon morphology distribution, consistent with another murine model after VCR treatment [57], though Geisler et al. [34] report a right shift. Nonetheless, *Sarm1* KO mitigated morphological changes in morphology suggesting its protective effect from VCR-induced alterations.

While VCR directly affects the peripheral nerves, it does not efficiently cross the blood-brain barrier [58] so that the nature of neurotoxicity in the brain is unclear. As the MRI data showed volume improvement for VCR-treated mice after deletion of *Sarm1*, we sought to determine whether knockout of *Sarm1* similarly altered the distribution of axons in the brain. We therefore conducted identical TEM analyses in the brain, taking the corpus callosum—the largest white matter tract—to be representative (Fig. 6G–J). Like the peripheral nerves, VCR treatment in wildtype mice resulted in an altered axon and myelin size distribution (Fig. 6K). However, unlike the peripheral nerves, *Sarm1* KO did not appear to protect axons in the corpus callosum as VCR altered axon morphology similar to wildtype mice. Both myelin and axon area distribution were similarly altered by VCR for both wildtype and *Sarm1* KO mice (Fig. 6K and L). This indicates that *Sarm1* KO does not directly protect axons in the brain from VCR-induced change, but that volume improvements observed by MRI are more likely secondary to effects of VCR peripherally, such as the prevention of VIPN. There were no significant differences in G-ratios between treatment groups and genotype (Supplementary Fig. 4). We also note that tissues were collected from a relatively small number of mice (*n* = 4–5 mice per group), so results should be considered preliminary.

## Discussion

Characterization of neurotoxicity associated with VCR has traditionally been focused on acute effects evident in the peripheral nerves, with the result that its potential occurrence in the brain has been overlooked. This study highlights the potential for VCR brain toxicity in a mouse model. Since VCR is a key element in ALL therapy that is administered at every treatment stage [5, 59], it is essential to determine if it could play a role in the development of neurocognitive deficits among survivors of childhood ALL. This study corroborates our previous findings that VCR causes widespread brain volume deficits [19] and further determines that disruption of the axon degeneration regulator gene, *Sarm1*, prevents these neuroanatomical deficits. Since corpus callosum axonal changes after VCR treatment were not protected by *Sarm1* KO, this indicates that SARM1-dependent processes may not occur directly in the brain. These results suggest that peripheral toxicities, such as VIPN, may be an important contributor of VCR-induced brain toxicities, indicating that targeting SARM1-mediated peripheral neuropathy may also help mitigate neurocognitive deficits in patients treated with VCR.

Consistent with previous work [19], we observed extensive VCR-induced volume loss across the brain, affecting both grey and white matter. The widespread volume reduction from VCR treatment in our study mirrors the phenotype also observed in ALL survivors, where both grey matter and white matter alterations in childhood ALL survivors have been reported [60–63]. For instance, a study of 71 ALL survivors exhibited 6% smaller white matter and 5% smaller grey matter volumes relative to controls [20]. Although VCR does not cross the blood-brain barrier efficiently, it is not uncommon for systemic toxicities to be linked with neurocognitive deficits [64]. One prime example is doxorubicin, which also does not cross the blood-brain barrier but is widely considered an important cause of cognitive deficits in breast cancer survivors [65, 66]. VCR and doxorubicin, like other cytotoxic drugs, have overlapping systemic side effects, including organ toxicities and peripheral inflammation [67–70]. Moreover, vinca alkaloids specifically have also drawn recent attention to being linked to increased frailty and accelerated biological aging [71]. The hallmark systemic side effect of VCR is the development of VIPN. Our results highlight that reduced peripheral axon degeneration in *Sarm1* KO mice shortly after VCR treatment is associated with better long-term outcomes in the brain by early adulthood (P63). This increases motivation to carefully manage VIPN, as long-term consequences may be greater than previously appreciated.

The longitudinal nature of the current study allowed us to evaluate both changes a few days following VCR treatment and long-term outcomes. MRI revealed that the VCR-induced brain volume deficits appeared soon after treatment (at childhood equivalent age) and then persisted into young adulthood in mice. *Sarm1* KO attenuated the volume loss early after treatment. Limiting this early volume loss is likely to be one important strategy for managing long-term brain toxicity. This early period post-treatment coincides with the acute manifestation of peripheral neuropathy symptoms [72]. Future experiments using the Cre-LoxP system can induce timed knockouts at specific time points to determine whether the acute rescue observed in *Sarm1* knockout mice is necessary for long-term recovery [73, 74]. We also observed that *Sarm1* KO enabled an improved growth and/or subsequent normalization of volume in early adulthood, such as in the dentate gyrus and amygdala. These results indicate that manipulating SARM1 not only mitigates early brain injury from the VCR effect but also promotes significant recovery in specific structures. The dentate gyrus of the hippocampus is one of the few places in the brain where neurogenesis occurs postnatally but can be disrupted by chemotherapy treatment [75]. Decreases in dentate gyrus volumes have been associated with impaired working memory and learning in childhood ALL survivors [76]. Similarly, smaller amygdala volumes have been linked to increased stress [77] and reduced working memory [78] in cancer survivors. Improved morphological outcomes in these structures by targeting SARM1 may also ameliorate neurocognitive deficits in survivors.

Although we did observe VCR-induced changes in the axonal population of the corpus callosum at P23 with TEM, our findings suggest that SARM1 had little impact on this change for axons within the brain, in contrast to the distal sciatic nerve where changes were eliminated. Importantly, this suggests that VCR-induced brain toxicity, including in the corpus callosum, occurs via a different mechanism than toxicity to peripheral nerves. This may also be reflected in the trends of VCR-induced mild alterations in FA and RD observed at P63 for both genotypes, suggesting that *Sarm1* knockout does not change brain axon sensitivity to VCR toxicity. Similarly, van der Plas et al. [20] reported no significant DTI metric differences between ALL survivors and controls after FDR correction, though they also noted trends toward reduced FA and increased RD in specific white matter regions. Nonetheless, long-term DTI changes may still be expected as other studies in pediatric ALL survivors have reported lower FA and increased directional diffusivity in white matter tracts [63, 79–81]. The lack of correlation between volumetric changes and diffusion metrics in our data, consistent with findings by van der Plas et al. [20], suggest volumetric alterations occur with limited microstructural alterations. While this suggests that assessing brain morphology may provide a more sensitive measure of treatment-related effects, we note that diffusion measurements were only conducted at endpoint in our study. Longitudinal diffusion measurements might provide additional insights or reveal early indicators of pathology that resolve over time.

Based on longitudinal MRI, *Sarm1* knockout mitigated neuroanatomical deficits from VCR treatment. It may be that manifestation of VIPN produces factors with downstream consequences for the brain. Indeed, several studies have found associations between chemotherapy-induced peripheral neuropathy and cognitive outcomes [36, 82, 83]. For example, Varedi et al. [84] found that the severity of peripheral neuropathy symptoms correlated with impaired visual-motor processing speeds in childhood ALL survivors. Patients who experienced neuropathic pain had a higher frequency of learning impairments post-chemotherapy [36]. Williams et al. [85] further showed that peripheral neuropathies are associated with future impairments in processing speed, sustained attention, verbal recall, and working memory in long-term pediatric ALL survivors. Proposed overlapping mechanisms linking peripheral nerve damage to brain toxicity include psychological distress [86, 87], direct afferent nerve hyperexcitability to the brain [88], and inflammation [89–91]. In the context of the latter, the potential role of the pro-inflammatory cytokine IL-1β is of particular interest, due to its role in mediating peripheral nerve injury, its upregulation in blood plasma, and the central nervous system [92–95]. Elevations of IL-1β in cerebrospinal fluid and the brain have also been associated with neurocognitive impairment [93]. While pain may contribute to cognitive impairment, Dimitrov et al. [96] found that impaired neurogenesis persisted even when alleviating mechanical allodynia in mice.

Our findings using a constitutive knockout of Sarm1 broadly implicates SARM1 in mediating VCR-induced brain toxicity. Although the EM and DTI results suggest that VCR impacts the peripheral axons differently from axons in the brain, an alternative strategy is necessary to elucidate SARM1 mechanisms specific to either the peripheral nervous system (PNS) or central nervous system (CNS). One approach would be to develop a conditional *Sarm1* knockout mouse model that targets the PNS and CNS in turn, such as through the Cre-loxP recombinase system [74]. For instance, if Sarm1 knockout in only PNS neurons produced a rescue effect similar to the constitutive Sarm1 knockouts, then it would strongly suggest that peripheral neuropathy does indeed precede CNS toxicity. Given the possibility that even trace levels of VCR could influence the brain, using a brain specific conditional Sarm1 knockout would allow direct assessment of VCR’s effects on central axons. However, in practice, finding promoters that differentiate between the CNS and PNS is difficult: promoters like *Camk2a* and *Emx1* are highly expressed in the brain but may still be expressed, albeit in smaller concentrations, in the PNS [97, 98]. An alternative approach to primarily target the PNS would be administer a SARM1 inhibitor that does not efficiently cross the blood-brain barrier. Furthermore, SARM1 inhibitors offer the advantage of clinical translatability, representing an exciting prospect given the lack of current treatments that directly target axon degeneration [99–101].

In summary, the results demonstrate that the deletion of *Sarm1* preserves peripheral axon health and enhances brain outcomes as measure by structural MRI in a pediatric mouse model of VCR treatment. While the mechanisms behind these improvements require further investigation, this study adds further evidence to support a link between peripheral neuropathy and neurocognitive deficits in pediatric ALL survivors. These findings provide further evidence that targeting SARM1 could be a promising approach for ameliorating both VIPN and improving neurocognitive outcomes in pediatric patients undergoing VCR treatment.

## Supplementary Information

Below is the link to the electronic supplementary material.

Supplementary Material 1

## Data Availability

Structural MR images and their segmentations for volumetric analysis have been archived in Zenodo (10.5281/zenodo.15750286). Instructions for running MAGeT and the Pydpiper toolkit can be found on GitHub (https://github.com/Mouse-Imaging-Centre/pydpiper/).

## Abbreviations

ALL: Acute lymphoblastic leukemia
DTI: Diffusion tensor imaging
FA: Fractional anisotropy
MD: Mean diffusivity
RD: Radial diffusivity
AD: Axial diffusivity
VCR: Vincristine
VIPN: Vincristine-induced peripheral neuropath
KO: Knockout
WT: Wildtype
