# Supplementary material for: Vincristine-induced brain toxicity is reduced with prevention of peripheral axon degeneration in Sarm1 knockout mice
Source: Acta Neuropathol Commun. 2025 Dec 19;13:254. doi: 10.1186/s40478-025-02171-0 (PMC12717744; doi:10.1186/s40478-025-02171-0)
Supplement: Supplementary file 1 — Supplementary Material 1 [file 40478_2025_2171_MOESM1_ESM.docx]

**Supplementary Material**

**Methods**

*Statistical analysis*

The linear mixed effects model for fitting the volumes is defined as a combination of intercepts, linear slopes, and a random effect term:

$$Volume=\beta_{0}+\beta_{1\left( \mathrm{Timepoint} \right)}+\beta_{2\left( VCR;t>17 \right)}+\beta_{3\left( SARM1 \right)}+\beta_{4\left( VCR;SARM1;t>17 \right)}$$

$${+\beta}_{5\left( VCR;t>17 \right)}\left( t-t_{o} \right)+\beta_{6\left( SARM1 \right)}\left( t-t_{o} \right)+\beta_{7\left( VCR;SARM1;t>17 \right)}\left( t-t_{o} \right)+\gamma_{\mathrm{MouseID}}$$

Random effects ($\gamma_{MouseID}$) were assigned by individual mouse ID and was common across all timepoints. The time $t_{o}$ is the reference timepoint in which the model is centered around to determine the effect at a particular timepoint (e.g., $t_{o}=23$ to obtain the vincristine (VCR) effect at P23). The term representing the volume at each timepoint, $\beta_{1\left( \mathrm{Timepoint} \right)}$, consists of individual coefficients for P14, P23, P42, and P63. Coefficients with the VCR effect is set to zero for timepoints before treatment (P17). The symbol “:” indicates an interaction term. Statistical analysis was performed using a linear-mixed models from the lme4 package in R.^1^

**Reference**

1. Bates D, Mächler M, Bolker B, Walker S. Fitting Linear Mixed-Effects Models Using **lme4**. *J Stat Soft*. 2015;67(1). doi:10.18637/jss.v067.i01

**Supplementary Figures**

**
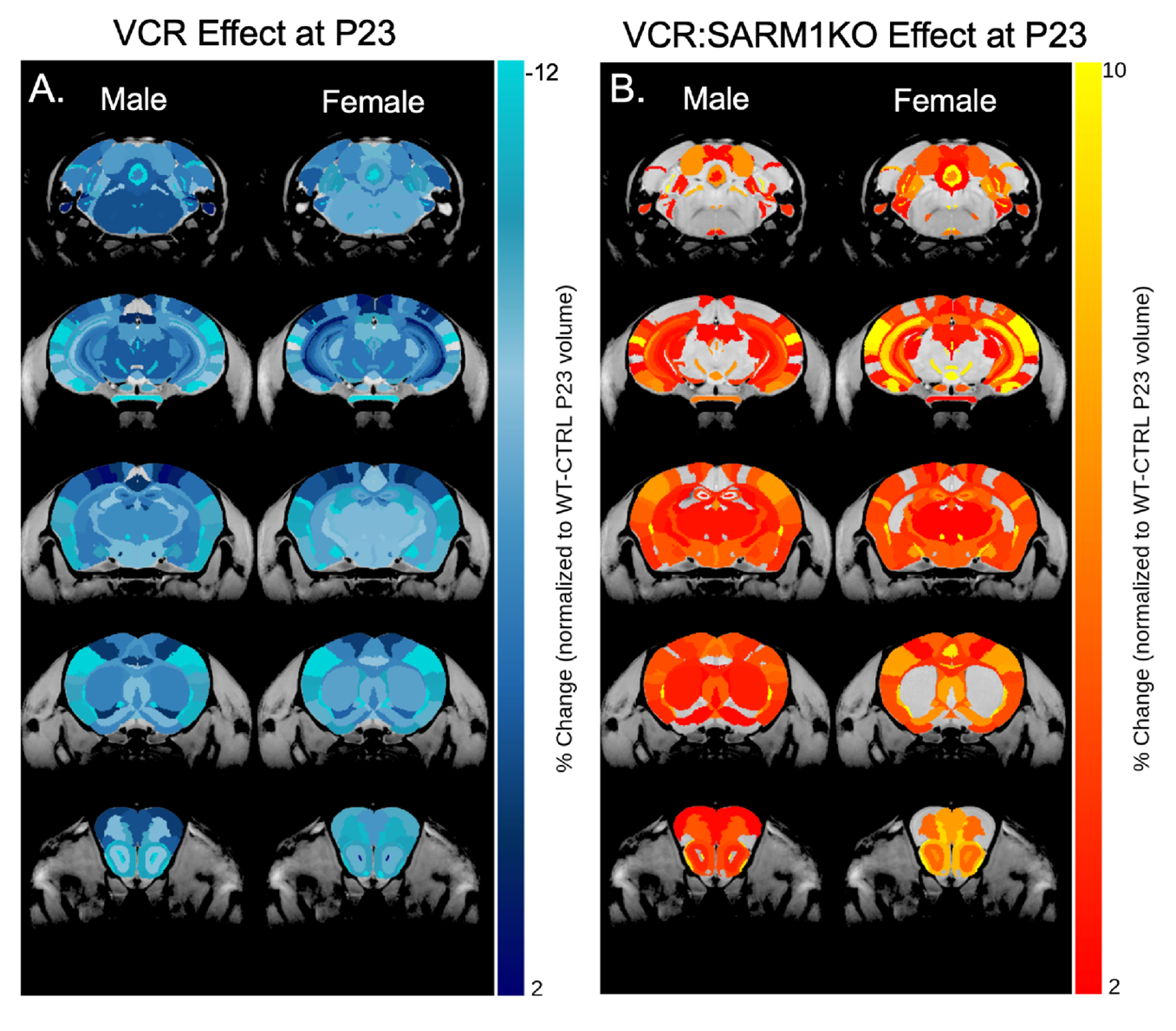
**

**Supplementary Figure 1:** The neuroanatomical plots of the VCR and VCR:SARM1KO effects with sex evaluated using a separate linear mixed-effect model. Highlighted structures represent regions that exhibit at least a 2% change, though none reached statistically significant after FDR correction (q>0.1). (A) The VCR effect at P23 on males and females separately shows similar regions affected. (B) Similar regions were also rescued from VCR at P23 due to SARM1KO.


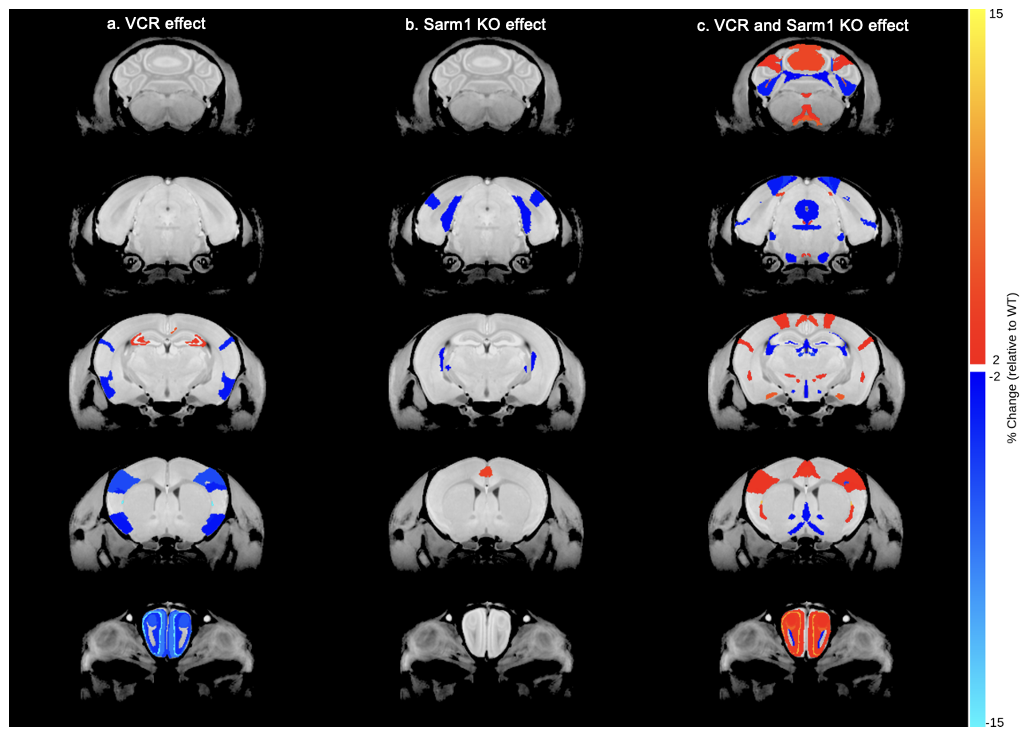


**Supplementary Figure 2:** The neuroanatomical plots of the VCR, *Sarm1* KO, and VCR-*Sarm1* KO effects on relative structural brain volumes at P23. A) The VCR effect on the brain (q<0.1) disproportionately reduces the olfactory bulb relative volumes while the hippocampal regions are increased. (B) Minimal effects on relative volumes of *Sarm1* KO in saline-treated mice, with few structures affected (p<0.05, uncorrected). No structures were considered significant at q<0.1. (C) The effect of VCR-*Sarm1* KO interaction on relative volumes qualitatively demonstrate a mitigation in VCR effect (p<0.05, uncorrected). No structures were considered significant at q<0.1.


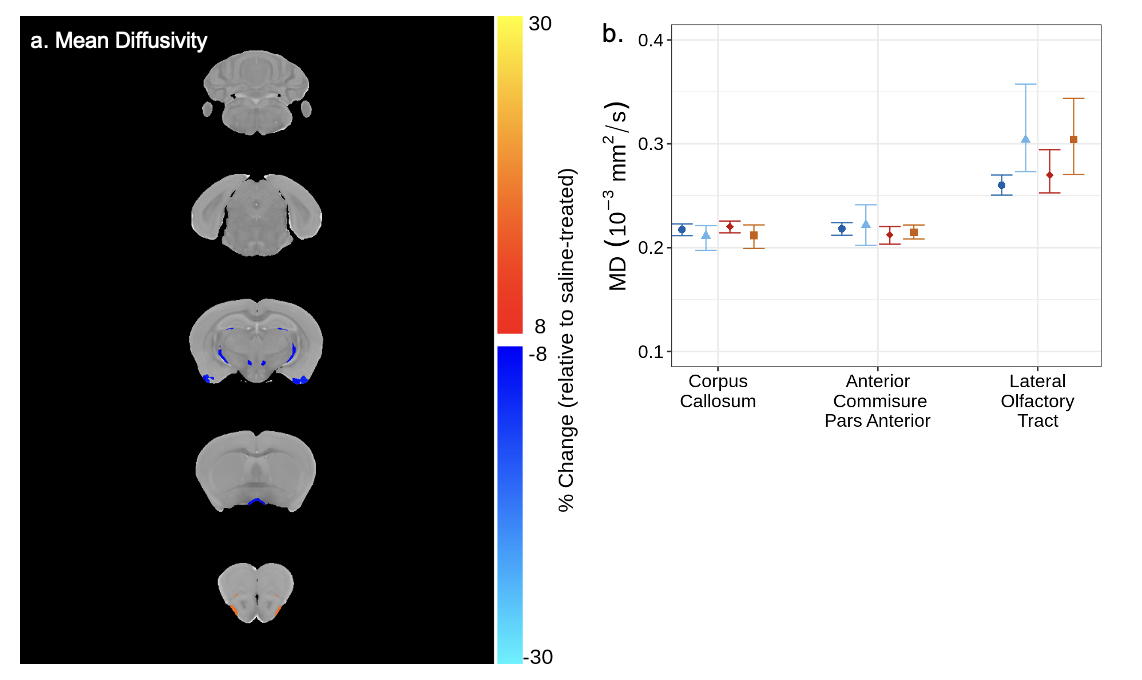


**Supplementary Figure 3:** The VCR effect on MD in wildtype and *Sarm1* KO mice at P63. (A) Neuroanatomical maps highlighting regions that exhibited a minimum magnitude of 8% change in MD. No changes were considered statistically significant (q>0.1). (B) The MD between treatment and genotype groups in several white matter structures. Points and whiskers represent mean structure volume and bootstrapped 95% confidence intervals, respectively.


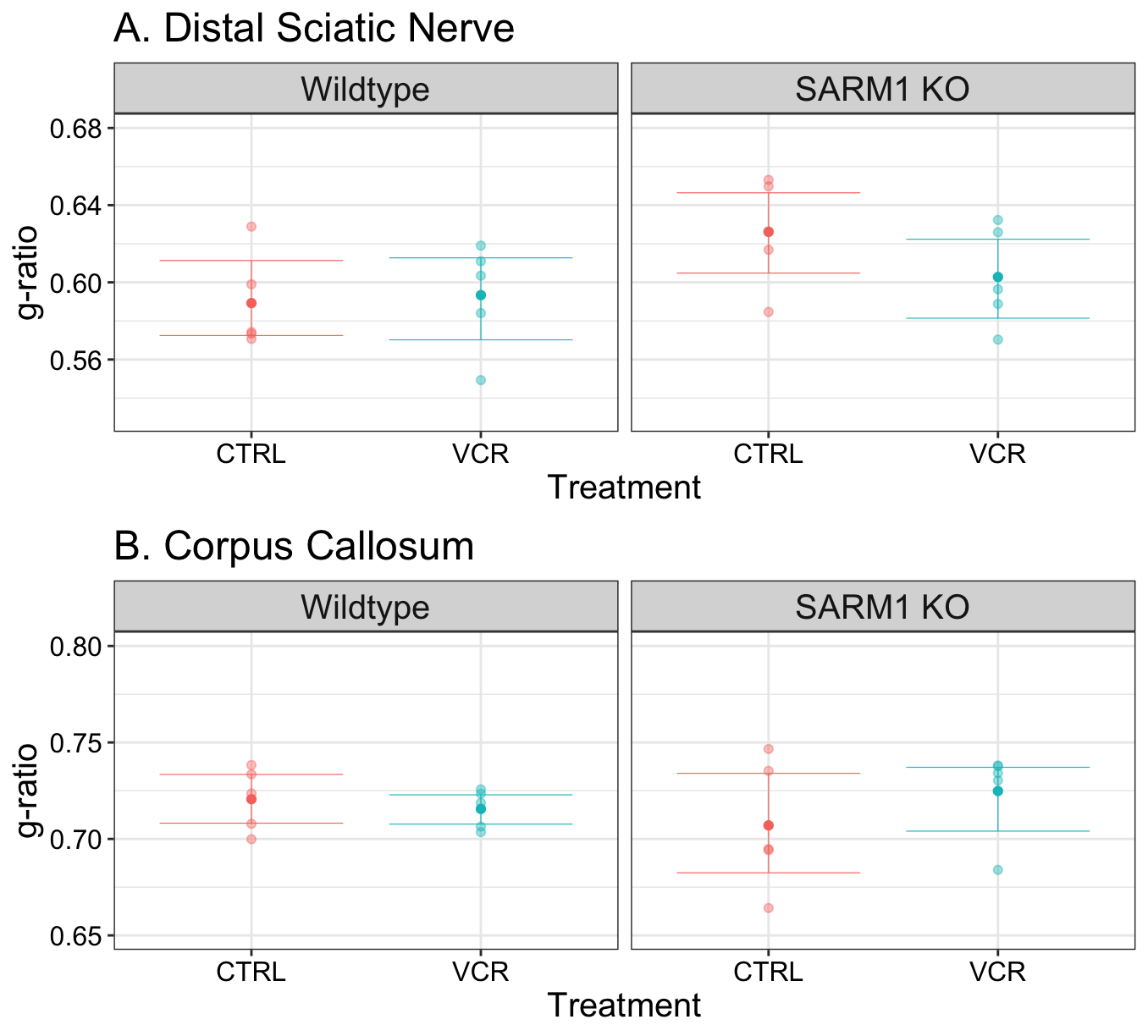


**Supplementary Figure 4:** The g-ratio for the distal sciatic nerve and corpus callosum between treatment groups for each genotype. No significant differences were found in g-ratio between treatment nor genotype.

**Supplementary Tables**

| **Timepoint**  **Group** | **14** | **23** | **42** | **63** |
| --- | --- | --- | --- | --- |
| CTRL Wildtype | 14 (7) | 14 (7) | 12 (6) | 14 (7) |
| VCR Wildtype | 16 (7) | 15 (7) | 13 (5) | 13 (5) |
| CTRL SARM1 KO | 15 (6) | 14 (5) | 11 (5) | 15 (6) |
| VCR SARM1 KO | 16 (8) | 15 (7) | 13 (7) | 16 (8) |

**Supplementary Table 1:** Mouse numbers for each group and imaging timepoint. The number of males is indicated in parantheses.

| **Structure** | **VCR** | | **SARM1 KO** | |  | | **VCR and SARM1 KO** | |
| --- | --- | --- | --- | --- | --- | --- | --- | --- |
|  | **% volume  change** | **Uncorrected  p-value** | **% volume  change** | **Uncorrected  p-value** | **% volume  change** | **% rescue (vs. VCR wildtype)** | | **Uncorrected  p-value** |
| amygdala | -5.51 | 0.001^†^ | -1.33 | 0.373 | 3.80 | 68.9 | | 0.043 |
| anterior_commissure_pars_anterior | -9.99 | <0.001^†^ | 0.43 | 0.838 | 2.18 | 21.9 | | 0.434 |
| anterior_commissure_pars_posterior | -7.21 | 0.023^†^ | -2.03 | 0.404 | 4.62 | 64.1 | | 0.229 |
| basal_forebrain | -7.74 | <0.001^†^ | -0.64 | 0.685 | 4.20 | 54.3 | | 0.011 |
| bed_nucleus_of_stria_terminalis | -8.73 | <0.001^†^ | -1.65 | 0.378 | 5.02 | 57.5 | | 0.013 |
| cerebellar_peduncle_inferior | -6.67 | 0.001^†^ | -2.48 | 0.139 | 2.24 | 33.6 | | 0.326 |
| cerebellar_peduncle_middle | -7.33 | <0.001^†^ | -0.04 | 0.981 | 1.08 | 14.7 | | 0.642 |
| cerebellar_peduncle_superior | -7.54 | 0.001^†^ | -3.09 | 0.105 | 1.20 | 15.9 | | 0.663 |
| cerebral_aqueduct | -9.28 | 0.020^†^ | 0.67 | 0.870 | 3.79 | 40.8 | | 0.417 |
| cerebral_peduncle | -7.16 | <0.001^†^ | 0.84 | 0.579 | 2.33 | 32.5 | | 0.168 |
| colliculus_inferior | -6.02 | <0.001^†^ | -0.40 | 0.802 | 4.48 | 74.4 | | 0.007^†^ |
| colliculus_superior | -6.79 | <0.001^†^ | 1.62 | 0.293 | 2.23 | 32.8 | | 0.154 |
| corpus_callosum | -6.16 | <0.001^†^ | 0.74 | 0.650 | 2.32 | 37.7 | | 0.142 |
| corticospinal_tract_pyramids | -6.40 | 0.009^†^ | -1.80 | 0.350 | 3.30 | 51.7 | | 0.262 |
| cuneate_nucleus | -5.81 | 0.108 | 3.65 | 0.244 | 1.29 | 22.2 | | 0.765 |
| facial_nerve_cranial_nerve_7 | -7.05 | 0.065^†^ | -3.02 | 0.275 | 5.69 | 80.8 | | 0.228 |
| fasciculus_retroflexus | -5.41 | 0.031^†^ | -0.31 | 0.882 | -1.93 | 0.0 | | 0.519 |
| fimbria | -7.16 | <0.001^†^ | 1.18 | 0.666 | 0.82 | 11.4 | | 0.676 |
| fornix | -6.98 | 0.004^†^ | 0.34 | 0.867 | 1.59 | 22.8 | | 0.576 |
| fourth_ventricle | -9.68 | <0.001^†^ | -2.99 | 0.132 | 6.69 | 69.1 | | 0.026 |
| fundus_of_striatum | -11.39 | 0.011^†^ | 1.25 | 0.709 | 8.04 | 70.6 | | 0.141 |
| globus_pallidus | -6.59 | <0.001^†^ | -1.59 | 0.389 | 3.57 | 54.2 | | 0.089 |
| habenular_commissure | -14.34 | 0.022^†^ | 0.36 | 0.957 | 7.44 | 51.9 | | 0.309 |
| hypothalamus | -7.90 | <0.001^†^ | -1.62 | 0.210 | 3.89 | 49.2 | | 0.004^†^ |
| inferior_olivary_complex | -14.14 | 0.002^†^ | 2.79 | 0.407 | 8.51 | 60.2 | | 0.123 |
| internal_capsule | -7.10 | <0.001^†^ | -3.16 | 0.084 | 4.11 | 57.9 | | 0.044 |
| interpedunclar_nucleus | -6.48 | 0.002^†^ | -1.93 | 0.286 | 8.23 | 100.0 | | 0.001^†^ |
| lateral_olfactory_tract | -13.76 | <0.001^†^ | -1.69 | 0.421 | 7.34 | 53.3 | | 0.017 |
| lateral_septum | -7.28 | <0.001^†^ | 0.01 | 0.994 | 4.35 | 59.7 | | 0.019 |
| lateral_ventricle | -6.08 | 0.009^†^ | 1.05 | 0.823 | 3.07 | 50.5 | | 0.251 |
| mammillary_bodies | -10.38 | <0.001^†^ | -1.50 | 0.486 | 7.73 | 74.5 | | 0.009 |
| mammilothalamic_tract | -13.22 | <0.001^†^ | 0.72 | 0.815 | 7.27 | 55.0 | | 0.105 |
| medial_lemniscus_medial_longitudinal_fasciculus | -9.89 | <0.001^†^ | -2.41 | 0.161 | 6.77 | 68.5 | | 0.012 |
| medial_septum | -4.46 | 0.011^†^ | 0.18 | 0.916 | -0.45 | 0.0 | | 0.825 |
| medulla | -6.72 | <0.001^†^ | -1.15 | 0.366 | 1.70 | 25.3 | | 0.195 |
| midbrain | -4.84 | <0.001^†^ | -0.38 | 0.766 | 0.58 | 11.9 | | 0.619 |
| nucleus_accumbens | -4.72 | 0.003^†^ | 1.50 | 0.383 | 1.02 | 21.7 | | 0.581 |
| olfactory_peduncle | -9.40 | <0.001^†^ | 0.38 | 0.831 | 4.76 | 50.6 | | 0.012 |
| olfactory_tubercle | -7.75 | <0.001^†^ | 0.27 | 0.884 | 2.31 | 29.8 | | 0.276 |
| optic_tract | -8.84 | <0.001^†^ | -0.82 | 0.622 | 5.46 | 61.8 | | 0.006^†^ |
| periaqueductal_grey | -5.36 | <0.001^†^ | 1.18 | 0.449 | 0.72 | 13.4 | | 0.652 |
| pons | -5.28 | <0.001^†^ | -1.01 | 0.457 | 1.89 | 35.8 | | 0.136 |
| pontine_nucleus | -8.69 | <0.001^†^ | 0.91 | 0.647 | 1.67 | 19.3 | | 0.471 |
| posterior_commissure | -8.89 | <0.001^†^ | 0.10 | 0.959 | 3.17 | 35.6 | | 0.281 |
| pre_para_subiculum | -3.10 | 0.035^†^ | -2.28 | 0.197 | 1.79 | 57.8 | | 0.296 |
| stria_medullaris | -6.82 | <0.001^†^ | -1.38 | 0.426 | 1.77 | 25.9 | | 0.421 |
| stria_terminalis | -9.30 | <0.001^†^ | -3.11 | 0.106 | 3.59 | 38.6 | | 0.121 |
| striatum | -6.06 | <0.001^†^ | -0.36 | 0.788 | 2.15 | 35.5 | | 0.131 |
| subependymale_zone_rhinocele | -5.77 | 0.147 | -1.22 | 0.697 | 4.96 | 86 | | 0.304 |
| superior_olivary_complex | -4.69 | 0.031^†^ | 2.82 | 0.138 | 0.16 | 3.5 | | 0.949 |
| thalamus | -7.00 | <0.001^†^ | -0.13 | 0.923 | 2.55 | 36.4 | | 0.035 |
| third_ventricle | -5.39 | 0.079^†^ | 0.66 | 0.852 | 2.75 | 51 | | 0.444 |
| ventral_tegmental_decussation | -1.47 | 0.663 | -5.73 | 0.034 | 4.19 | 100 | | 0.306 |
| lobules_1_2_lingula_and_central_lobule_ventral | -4.59 | 0.010^†^ | -2.03 | 0.397 | 2.61 | 56.9 | | 0.206 |
| lobule_3_central_lobule_dorsal | -5.53 | <0.001^†^ | -0.26 | 0.884 | 5.81 | 100 | | 0.001^†^ |
| lobules_4_5_culmen_ventral_and_dorsal | -7.31 | <0.001^†^ | -1.12 | 0.566 | 7.89 | 100 | | 0.001^†^ |
| lobule_6_declive | -5.94 | 0.004^†^ | 0.78 | 0.731 | 1.60 | 26.9 | | 0.506 |
| lobule_7_tuber_or_folium | -9.77 | <0.001^†^ | 1.04 | 0.718 | 3.65 | 37.3 | | 0.107 |
| lobule_8_pyramis | -4.39 | 0.033^†^ | 2.05 | 0.371 | -2.78 | 0 | | 0.248 |
| lobule_9_uvula | -3.78 | 0.047^†^ | 1.43 | 0.490 | 2.69 | 71.1 | | 0.229 |
| lobule_10_nodulus | -6.52 | 0.002^†^ | 2.22 | 0.261 | 4.26 | 65.3 | | 0.090 |
| anterior_lobule_lobules_4_5 | -5.95 | <0.001^†^ | -1.38 | 0.467 | 2.61 | 44 | | 0.206 |
| simple_lobule_lobule_6 | -7.27 | <0.001^†^ | -1.50 | 0.402 | 3.86 | 53.1 | | 0.066 |
| crus_1_ansiform_lobule_lobule_6 | -8.08 | <0.001^†^ | -2.38 | 0.148 | 5.12 | 63.4 | | 0.010 |
| crus_2_ansiform_lobule_lobule_7 | -5.40 | 0.003^†^ | -1.45 | 0.495 | 3.56 | 65.9 | | 0.087 |
| paramedian_lobule_lobule_7 | -4.88 | 0.001^†^ | 1.54 | 0.410 | -1.74 | 0 | | 0.324 |
| copula_pyramis_lobule_8 | -7.38 | 0.000^†^ | 1.15 | 0.516 | 2.16 | 29.3 | | 0.248 |
| flocculus_FL | -5.91 | 0.006^†^ | 0.21 | 0.936 | 1.94 | 32.8 | | 0.436 |
| paraflocculus_PFL | -3.25 | 0.103 | 5.39 | 0.096 | 3.87 | 100 | | 0.095 |
| trunk_of_arbor_vita | -5.66 | <0.001^†^ | 0.11 | 0.940 | 0.53 | 9.5 | | 0.778 |
| lobule_1_2_white_matter | -21.55 | <0.001^†^ | -1.74 | 0.721 | 11.00 | 51 | | 0.112 |
| lobule_3_white_matter | -10.62 | <0.001^†^ | -6.86 | 0.006 | 9.60 | 90.4 | | 0.008^†^ |
| trunk_of_lobules_1_3_white_matter | -5.97 | 0.093 | -0.91 | 0.747 | 2.59 | 43.4 | | 0.545 |
| lobules_4_5_white_matter | -7.53 | 0.008^†^ | -3.73 | 0.144 | 6.07 | 80.7 | | 0.071 |
| lobules_6_7_white_matter | -6.73 | 0.011^†^ | -0.58 | 0.817 | 5.88 | 87.4 | | 0.059 |
| lobule_8_white_matter | -22.28 | <0.001^†^ | -7.58 | 0.144 | 16.42 | 73.7 | | 0.031 |
| trunk_of_lobules_6_8_white_matter | -5.78 | 0.190 | -2.70 | 0.490 | 0.94 | 16.2 | | 0.858 |
| lobule_9_white_matter | -6.72 | 0.223 | -2.12 | 0.610 | 7.00 | 100 | | 0.299 |
| lobule_10_white_matter | -15.72 | 0.066^†^ | -9.40 | 0.145 | 13.66 | 86.9 | | 0.191 |
| anterior_lobule_white_matter | -10.40 | 0.012^†^ | -8.24 | 0.024 | 9.41 | 90.5 | | 0.056 |
| simple_lobule_white_matter | -14.34 | 0.002^†^ | -4.67 | 0.240 | 10.06 | 70.1 | | 0.063 |
| crus_1_white_matter | -10.93 | 0.010^†^ | -1.38 | 0.686 | 6.43 | 58.8 | | 0.204 |
| trunk_of_simple_and_crus_1_white_matter | -4.22 | 0.196 | -3.54 | 0.215 | 1.98 | 46.8 | | 0.612 |
| crus_2_white_matter | -13.53 | 0.012^†^ | -1.30 | 0.771 | 8.92 | 65.9 | | 0.165 |
| paramedian_lobule | -13.11 | 0.021^†^ | -4.59 | 0.351 | 10.29 | 78.5 | | 0.131 |
| trunk_of_crus_2_and_paramedian_white_matter | -7.29 | 0.011^†^ | -1.40 | 0.596 | 4.09 | 56 | | 0.229 |
| copula_white_matter | -1.08 | 0.804 | -4.69 | 0.209 | 5.64 | 100 | | 0.283 |
| paraflocculus_white_matter | -7.71 | 0.014^†^ | 0.18 | 0.950 | 7.80 | 100 | | 0.037 |
| flocculus_white_matter | -16.91 | 0.024^†^ | -8.57 | 0.211 | 18.35 | 100 | | 0.042 |
| dentate_nucleus | -7.80 | <0.001^†^ | -2.92 | 0.139 | 3.33 | 42.8 | | 0.193 |
| nucleus_interpositus | -8.54 | <0.001^†^ | -1.23 | 0.505 | 3.01 | 35.3 | | 0.231 |
| fastigial_nucleus | -6.88 | 0.006^†^ | -2.22 | 0.261 | 3.77 | 54.9 | | 0.207 |
| Cingulate_cortex_area_24a | -5.02 | 0.002^†^ | 1.67 | 0.454 | 3.10 | 61.8 | | 0.093 |
| Cingulate_cortex_area_24a.1 | -5.37 | 0.019^†^ | -0.46 | 0.838 | 3.01 | 56.2 | | 0.263 |
| Cingulate_cortex_area_24b | -6.56 | <0.001^†^ | 2.52 | 0.147 | 4.33 | 65.9 | | 0.050 |
| Cingulate_cortex_area_24b.1 | -5.05 | 0.069^†^ | -3.98 | 0.076 | 5.58 | 100 | | 0.096 |
| Cingulate_cortex_area_25 | -12.74 | <0.001^†^ | -1.91 | 0.427 | 8.82 | 69.2 | | 0.001^†^ |
| Cingulate_cortex_area_29a | -5.83 | 0.007^†^ | -1.59 | 0.443 | 6.04 | 100 | | 0.018 |
| Cingulate_cortex_area_29b | -6.51 | 0.004^†^ | 1.59 | 0.367 | 1.94 | 29.8 | | 0.473 |
| Cingulate_cortex_area_29c | -2.22 | 0.220 | -0.11 | 0.952 | 1.61 | 72.3 | | 0.453 |
| Cingulate_cortex_area_30 | -3.44 | 0.024^†^ | -0.63 | 0.666 | 2.86 | 83.4 | | 0.111 |
| Cingulate_cortex_area_32 | -6.13 | 0.002^†^ | 2.54 | 0.226 | 6.85 | 100 | | 0.003^†^ |
| Amygdalopiriform_transition_area | -5.00 | 0.070^†^ | -1.88 | 0.431 | 2.36 | 47.2 | | 0.475 |
| Primary_auditory_cortex | -9.20 | 0.002^†^ | -2.44 | 0.259 | 5.66 | 61.5 | | 0.113 |
| Secondary_auditory_cortex_dorsal_area | -8.09 | 0.002^†^ | -4.97 | 0.012 | 4.68 | 57.8 | | 0.138 |
| Secondary_auditory_cortex_ventral_area | -13.45 | <0.001^†^ | 0.07 | 0.978 | 7.52 | 55.9 | | 0.026 |
| Caudomedial_entorhinal_cortex | -4.78 | 0.003^†^ | -1.23 | 0.461 | -0.40 | 0 | | 0.833 |
| Cingulum | -5.89 | 0.002^†^ | 1.49 | 0.441 | 2.96 | 50.3 | | 0.180 |
| Claustrum | -16.78 | <0.001^†^ | -2.22 | 0.479 | 7.50 | 44.7 | | 0.102 |
| Cortex_amygdala_transition_zones | -5.17 | 0.040^†^ | -1.31 | 0.530 | 2.69 | 51.9 | | 0.373 |
| Claustrum_dorsal_part | -26.12 | <0.001^†^ | -3.51 | 0.470 | 16.09 | 61.6 | | 0.037 |
| Dorsal_nucleus_of_the_endopiriform | -11.51 | <0.001^†^ | -2.57 | 0.152 | 6.83 | 59.3 | | 0.007^†^ |
| Dorsal_intermediate_entorhinal_cortex | -6.20 | 0.003^†^ | -1.33 | 0.438 | 1.70 | 27.4 | | 0.487 |
| Dorsolateral_entorhinal_cortex | -7.86 | <0.001^†^ | -0.14 | 0.948 | -0.23 | 0 | | 0.927 |
| Dorsolateral_orbital_cortex | -6.52 | 0.012^†^ | -0.03 | 0.988 | 2.91 | 44.6 | | 0.344 |
| Dorsal_tenia_tecta | -8.31 | 0.001^†^ | -0.54 | 0.821 | 5.24 | 63 | | 0.086 |
| Ectorhinal_cortex | -6.86 | 0.015^†^ | -0.09 | 0.971 | 0.79 | 11.5 | | 0.815 |
| Frontal_cortex_area_3 | -12.46 | <0.001^†^ | 1.69 | 0.541 | 1.92 | 15.4 | | 0.655 |
| Frontal_association_cortex | -5.88 | 0.010^†^ | 0.58 | 0.765 | 3.94 | 67 | | 0.146 |
| Intermediate_nucleus_endopiriform_claustrum | -14.62 | <0.001^†^ | -1.66 | 0.512 | 5.95 | 40.7 | | 0.089 |
| Insular_region_not_subdivided | -9.19 | <0.001^†^ | -0.55 | 0.733 | 3.39 | 36.9 | | 0.083 |
| Lateral_orbital_cortex | -9.15 | <0.001^†^ | -0.34 | 0.848 | 5.97 | 65.3 | | 0.003^†^ |
| Lateral_parietal_association_cortex | -7.51 | 0.205 | -2.28 | 0.608 | 1.12 | 14.9 | | 0.878 |
| Primary_motor_cortex | -5.31 | <0.001^†^ | 0.25 | 0.867 | 4.27 | 80.4 | | 0.013 |
| Secondary_motor_cortex | -4.50 | 0.003^†^ | 1.51 | 0.289 | 3.15 | 70.1 | | 0.075 |
| Medial_entorhinal_cortex | -9.55 | <0.001^†^ | -0.53 | 0.800 | 1.73 | 18.1 | | 0.553 |
| Medial_orbital_cortex | -6.35 | 0.005^†^ | -1.74 | 0.446 | 6.39 | 100 | | 0.017 |
| Medial_parietal_association_cortex | 1.22 | 0.764 | 1.44 | 0.648 | 2.12 | 0 | | 0.667 |
| Piriform_cortex | -10.23 | <0.001^†^ | -0.13 | 0.925 | 3.17 | 31 | | 0.065 |
| Posterolateral_cortical_amygdaloid_area | -9.65 | 0.005^†^ | -0.72 | 0.794 | 2.31 | 23.9 | | 0.571 |
| Posteromedial_cortical_amygdaloid_area | -8.21 | 0.003^†^ | -1.16 | 0.638 | 6.92 | 84.2 | | 0.032 |
| Perirhinal_cortex | -7.81 | <0.001^†^ | 0.37 | 0.849 | 1.22 | 15.6 | | 0.652 |
| Parietal_cortex_posterior_area_rostral_part | -8.55 | 0.514 | -10.69 | 0.268 | -1.46 | 0 | | 0.928 |
| Rostral_amygdalopiriform_area | -6.92 | 0.111 | 0.66 | 0.846 | 1.23 | 17.7 | | 0.815 |
| Primary_somatosensory_cortex | -12.36 | <0.001^†^ | -0.35 | 0.846 | 6.19 | 50.1 | | 0.010 |
| Primary_somatosensory_cortex_barrel_field | -4.75 | 0.004^†^ | 0.12 | 0.939 | 4.57 | 96.1 | | 0.020 |
| Primary_somatosensory_cortex_dysgranular_zone | -26.31 | <0.001^†^ | -2.13 | 0.692 | 4.90 | 18.6 | | 0.575 |
| Primary_somatosensory_cortex_forelimb_region | -5.49 | 0.005^†^ | -0.32 | 0.846 | 5.52 | 100 | | 0.017 |
| Primary_somatosensory_cortex_hindlimb_region | -6.47 | 0.010^†^ | -0.42 | 0.841 | 5.84 | 90.3 | | 0.051 |
| Primary_somatosensory_cortex_jaw_region | -16.41 | <0.001^†^ | 0.44 | 0.901 | 1.29 | 7.8 | | 0.818 |
| Primary_somatosensory_cortex_shoulder_region | -10.82 | 0.221 | 2.77 | 0.670 | 15.16 | 100 | | 0.164 |
| Primary_somatosensory_cortex_trunk_region | -0.32 | 0.937 | -0.41 | 0.898 | 1.78 | 100 | | 0.721 |
| Primary_somatosensory_cortex_upper_lip_region | -11.34 | <0.001^†^ | -0.97 | 0.599 | 6.36 | 56.1 | | 0.004^†^ |
| Secondary_somatosensory_cortex | -8.74 | <0.001^†^ | -1.29 | 0.489 | 3.47 | 39.7 | | 0.118 |
| Temporal_association_area | -4.50 | 0.060^†^ | 0.21 | 0.918 | 0.72 | 16 | | 0.801 |
| Primary_visual_cortex | -4.09 | 0.082^†^ | 0.18 | 0.930 | -2.01 | 0 | | 0.475 |
| Primary_visual_cortex_binocular_area | -5.69 | 0.040^†^ | 0.40 | 0.850 | 1.18 | 20.7 | | 0.726 |
| Primary_visual_cortex_monocular_area | -6.93 | 0.013^†^ | -1.36 | 0.534 | -0.97 | 0 | | 0.774 |
| Secondary_visual_cortex_lateral_area | -3.65 | 0.102 | -2.80 | 0.113 | 0.99 | 27.1 | | 0.714 |
| Secondary_visual_cortex_mediolateral_area | -8.06 | 0.003^†^ | -1.81 | 0.413 | -2.77 | 0 | | 0.384 |
| Secondary_visual_cortex_mediomedial_area | -5.85 | 0.006^†^ | -1.32 | 0.445 | 0.38 | 6.5 | | 0.879 |
| Claustrum_ventral_part | -13.23 | <0.001^†^ | -3.66 | 0.155 | 8.22 | 62.1 | | 0.035 |
| Ventral_nucleus_of_the_endopiriform_claustrum | -11.45 | 0.004^†^ | -0.15 | 0.960 | 3.91 | 34.1 | | 0.409 |
| Ventral_intermediate_entorhinal_cortex | -9.86 | <0.001^†^ | -1.93 | 0.371 | 5.99 | 60.7 | | 0.041 |
| Ventral_orbital_cortex | -10.59 | <0.001^†^ | -1.23 | 0.551 | 7.40 | 69.9 | | 0.003^†^ |
| Ventral_tenia_tecta | -10.79 | 0.001^†^ | 3.32 | 0.336 | -3.19 | 0 | | 0.415 |
| CA10r | -4.42 | 0.009^†^ | -0.93 | 0.575 | 3.48 | 78.7 | | 0.081 |
| LMol | -5.08 | 0.002^†^ | -0.83 | 0.611 | 2.29 | 45.1 | | 0.241 |
| CA1Rad | -5.09 | 0.001^†^ | -1.00 | 0.549 | 3.56 | 69.9 | | 0.053 |
| CA2Py | -4.81 | 0.023^†^ | -2.12 | 0.258 | 3.54 | 73.6 | | 0.161 |
| CA20r | -7.68 | <0.001^†^ | -0.86 | 0.637 | 4.59 | 59.8 | | 0.041 |
| CA2Rad | -6.27 | <0.001^†^ | -1.13 | 0.543 | 4.05 | 64.6 | | 0.069 |
| CA3Py_Inner | -3.14 | 0.106 | -1.67 | 0.360 | 1.30 | 41.5 | | 0.572 |
| CA3Py_Outer | -5.01 | 0.003^†^ | 0.14 | 0.949 | 2.85 | 56.7 | | 0.146 |
| CA30r | -4.84 | <0.001^†^ | 0.45 | 0.829 | 2.86 | 59.2 | | 0.088 |
| CA3Rad | -4.49 | 0.003^†^ | -0.59 | 0.707 | 3.20 | 71.3 | | 0.069 |
| SLu | -5.35 | 0.003^†^ | -0.55 | 0.766 | 3.71 | 69.3 | | 0.082 |
| MoDG | -5.26 | <0.001^†^ | 0.03 | 0.983 | 1.85 | 35.2 | | 0.277 |
| GrDG | -5.09 | 0.002^†^ | -0.05 | 0.978 | 1.65 | 32.4 | | 0.397 |
| PoDG | -4.65 | 0.006^†^ | -0.08 | 0.967 | 1.07 | 22.9 | | 0.588 |
| CA1Py | -6.67 | 0.008^†^ | -0.79 | 0.727 | 4.84 | 72.5 | | 0.106 |
| Olfactory_bulb_glomerular_layer | -11.79 | <0.001^†^ | -1.07 | 0.655 | 0.52 | 4.4 | | 0.874 |
| Olfactory_bulb_external_plexiform_layer | -15.11 | <0.001^†^ | -2.75 | 0.161 | 4.07 | 26.9 | | 0.110 |
| Olfactory_bulb_mitral_cell_layer | -25.21 | <0.001^†^ | -0.40 | 0.919 | 11.59 | 46 | | 0.042 |
| Olfactory_bulb_internal_plexiform_layer | -24.04 | <0.001^†^ | 0.00 | 1.000 | 12.64 | 52.6 | | 0.032 |
| Olfactory_bulb_granule_cell_layer | -11.53 | <0.001^†^ | -1.27 | 0.532 | 5.58 | 48.4 | | 0.037 |
| Accessory_olfactory_bulb_glomerular  external_plexiform_and_mitral_cell_layer | -14.12 | <0.001^†^ | -2.48 | 0.237 | 6.20 | 43.9 | | 0.017 |
| Accessory_olfactory_bulb_granule_cell_layer | -10.55 | 0.003^†^ | -1.48 | 0.637 | 10.16 | 96.3 | | 0.015 |
| Anterior_olfactory_nucleus | -8.72 | <0.001^†^ | -0.18 | 0.927 | 4.20 | 48.2 | | 0.031 |
| subiculum | -5.22 | <0.001^†^ | -1.77 | 0.202 | 2.60 | 49.8 | | 0.087 |
| Medial_amygdala | -10.16 | <0.001 | -2.67 | 0.222 | 5.45 | 53.6 | | 0.049 |
| Medial_preoptic_nucleus | -14.84 | 0.007 | -2.98 | 0.477 | 10.33 | 69.6 | | 0.123 |
| Pituitary_gland | -15.98 | <0.001 | -4.59 | 0.153 | 7.46 | 46.7 | | 0.131 |

**Supplementary Table 2:** Segmented structures from the atlas with 183 labels. The percentage change of volumes at P23 are shown for the vincristine (VCR) effect, the SARM1 KO effect, and its interaction. The associated uncorrected p-values are shown. P-values that met the FDR of 10% are marked as ^†^. The percent rescue was calculated for each structure by dividing the amount of volume recovered relative to the VCR effect at P63 from the linear-mixed effects model. Values that exceeded 100% were set to 100 and negative values were set to 0.
